# A genome-wide association study in mice reveals a role for *Rhbdf2* in skeletal homeostasis

**DOI:** 10.1038/s41598-020-60146-8

**Published:** 2020-02-24

**Authors:** Roei Levy, Clemence Levet, Keren Cohen, Matthew Freeman, Richard Mott, Fuad Iraqi, Yankel Gabet

**Affiliations:** 1 Department of Anatomy and Anthropology, Tel Aviv University, Tel Aviv, 69978 Israel; 2 Department of Clinical Microbiology and Immunology, Sackler Faculty of Medicine, Tel Aviv University, Tel Aviv, 69978 Israel; 3 Dunn School of Pathology, South Parks Road, Oxford, OX1 3RE UK; 4 UCL Genetics Institute, University College London, Gower St., London, WC1E 6BT UK

**Keywords:** Computational models, Genome-wide association studies

## Abstract

Low bone mass and an increased risk of fracture are predictors of osteoporosis. Individuals who share the same bone-mineral density (BMD) vary in their fracture risk, suggesting that microstructural architecture is an important determinant of skeletal strength. Here, we utilized the rich diversity of the Collaborative Cross mice to identify putative causal genes that contribute to the risk of fractures. Using microcomputed tomography, we examined key structural features that pertain to bone quality in the femoral cortical and trabecular compartments of male and female mice. We estimated the broad-sense heritability to be 50–60% for all examined traits, and we identified five quantitative trait loci (QTL) significantly associated with six traits. We refined each QTL by combining information inferred from the ancestry of the mice, ranging from RNA-Seq data and published literature to shortlist candidate genes. We found strong evidence for new candidate genes, particularly *Rhbdf2*, whose close association with the trabecular bone volume fraction and number was strongly suggested by our analyses. We confirmed our findings with mRNA expression assays of *Rhbdf2* in extreme-phenotype mice, and by phenotyping bones of *Rhbdf2* knockout mice. Our results indicate that *Rhbdf2* plays a decisive role in bone mass accrual and microarchitecture.

## Introduction

Osteoporosis is characterized by a low bone mass and is associated with an increased risk of bone fractures. It is the most common bone disease, affecting nearly half the population over the age of 50 years in western countries^1^. The onset of osteoporosis results from peak values achieved at skeletal maturity, along with subsequent age- and menopause-related bone loss. Genetic factors play a significant role in determining the wide range of so-called “normal” peak bone mass. Quantitative measures of bone morphology are inherently complex traits; they are controlled by the cumulative effects and interactions of numerous genetic and environmental factors^2–4^.

Although genome-wide association studies (GWASs) in human populations have so far identified over 50 loci associated with bone mineral density (BMD)^5–11^, many other genes that have been experimentally associated with bone mass remain undetected in human cohorts^12–14^. This suggests that the BMD phenotype does not capture the full structural complexity of the bone^13^. GWASs have largely used areal bone mineral density (aBMD) as their bone phenotype. aBMD is measured by dual-energy X-ray absorptiometry (DXA). This is a two-dimensional projection that cannot measure the bone size, the shape of individual bone compartments (whether trabecular or cortical bone), or the underlying microstructure of the bone. Thus, it ignores important features potentially controlled by different genes. Indeed, a growing body of evidence favors distinguishing between cortical and trabecular bone, in light of genes that were observed to separately influence each bone type^9,10^. Importantly, a recent DXA-based GWAS, conducted with Collaborative Cross (CC; see below) mice, failed to find any heritability of aBMD^15^. Instead, using a similar mouse panel, we demonstrated that most trabecular microstructural parameters measured by micro-computed tomography (µCT) are moderately to highly heritable^16^.

Traditional peripheral quantitative CT (pQCT) can distinguish between cortical and trabecular bone compartments; however, it lacks the resolution necessary to detect microstructural differences. A recent report that utilized high-resolution pQCT (HR-pQCT) data in humans identified two novel bone-related loci that were invisible to DXA and pQCT-based GWAS^10^. Similar to HR-pQCT studies in humans, µCT scanning of rodents is used to better understand the genetic regulation of bone microstructural parameters and to distinguish genetic factors different from those previously identified for DXA-derived traits.

The CC mouse panel is designed to provide high-resolution analysis of complex traits, with special emphasis on traits relevant to human health^17,18^. This resource comprises a panel of recombinant inbred lines (RIL) descended from eight divergent strains of mice^19^. In contrast to commonly used laboratory mouse strains, the ancestry of the CC lines includes three wild-derived inbred strains that encompass genetic variations accumulated over ~1 million years^20^; more than 50 million single nucleotide polymorphisms segregate in founders of the CC. The high genetic diversity makes the panel potentially capable of identifying genes that would have remained anonymous in a population descended exclusively from strains derived from *Mus musculus domesticus*^21,22^. Here, we conducted a GWAS in a CC mouse cohort phenotyped by µCT, and as a result, we suggested *Rhbdf2* as a new genetic determinant of bone biology. We then analyzed the RNA expression patterns of selected candidate genes as well as knockout mice to further demonstrate the role of *Rhbdf2* in regulating bone density and microstructure.

## Results

### Population composition

To avoid false positive associations, we sampled from our pool of ~50 CC lineages, which included at least 30 new CC lines, and recorded the most common QTL peaks. Considering only these peaks as valid, we searched for the set that amplifies them the most, using a statistical approach aimed at rectifying the Winner’s Curse bias^23^; here, sets that reproduced the same signals were considered replicates^23^. This approach yielded a working cohort of 34 lines, of which only 20 lines were included in our previous report^16^. Notably, although a bigger cohort in terms of unique lineages may increase the power of association studies, it can also dilute the allelic pool due to the very nature of complex traits. This often occurs when a given phenotype is regulated by different sets of genes across different lineages. A recent study^24^ has shown that a CC panel with as few as 30 strains with sufficient replicates can reach a power of >80%. Our final population of 34 CC lines consisted of 174 mice: 71 females and 103 males; we examined the cortical and trabecular traits of the femoral bone.

### CC lines widely differ in their bone microarchitecture traits

**Figure 1:**
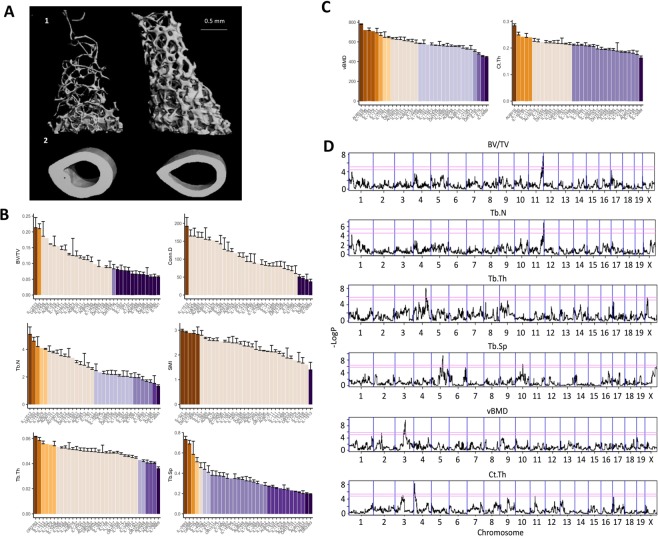
Phenotypic diversity in the CC panel and association test mapping. (**A**) µCT images of the trabecular (top) and cortical (bottom) bone of the femora of representative male CC mice. Top left: IL-4052; top right: IL-1513; bottom left: IL-785; bottom right: IL-2689. Distribution of trabecular (**B**) and cortical (**C**) traits across the CC lines. The x-axis represents the lines; the y-axis represents the trait means. (**B**) From top left, counter-clockwise: BV/TV (%), Tb.N (mm^−1^), Tb.Th (μm), Conn.D (mm^−3^), SMI, and Tb.Sp (mm). (**C**) Left, vBMD (mgHA/cm^3^); right, Ct.Th (mm). Color codes denote group lines that significantly differ from other groups. Lines are ordered differently between traits, in trait-specific descending order. See Table S3 for details. (**D**) Genome-wide association maps for the trabecular and cortical traits. The x-axis represents the position on the chromosome; the y-axis represents the –log*P* value of the association. The lower threshold represents the 95^th^ percentile of 200 simulations, and the top one represents the 99^th^ percentile. The loci above the 99% cut-off were further investigated. From top to bottom: BV/TV, Tb.N, Tb.Th, Tb.Sp, vBMD, and Ct.Th.

The morphometric parameters measured in the trabecular bone compartment included the trabecular bone volume fraction (BV/TV; %), the trabecular thickness (Tb.Th; µm), the trabecular number (Tb.N; mm^−1^), the trabecular connectivity density (Conn.D; mm^−3^), the trabecular structure model index (SMI), and the trabecular separation (Tb.Sp; mm). Cortical bone parameters consisted of volumetric bone mineral density (vBMD) and cortical thickness (Ct.Th) measured in the midshaft. The trabecular and cortical bone traits were approximately normally distributed; BV/TV ranged from 1.7 to 26% (mean = 10.2%), Tb.N from 0.52 to 6.11 mm^−1^ (mean = 2.7 mm^−1^), Tb.Th from 31 to 69 µm (mean = 47 µm), Conn.D from 10.9 to 268.3 mm^−3^ (mean = 104.2 mm^−3^), SMI from 0.6 to 3.3 (mean = 2.3), Tb.Sp from 0.16 to 0.7 mm (mean = 0.33 mm), Ct.Th from 0.14 to 0.29 mm (mean = 0.2 mm), and vBMD from 402.5 to 809.2 mgHA/cm^3^ (mean = 581.1 mgHA/cm^3^). Figure 1A shows µCT images taken from two mice with distinct cortical and trabecular characteristics. The mice exhibit visually highlighted differences in bone traits, presumably reflecting their genetic backgrounds. The color codes of the graphs in Fig. 1B,C represent Duncan’s least significance ranges (LSR), which indicate whether the mean value of a line or a group of lines, for a given trait, differs at a *P-*value <0.001 from any other group; this allows a visual representation of the heterogeneity among the lines. With 11 distinct groups, vBMD (Fig. 1C) is the most heterogeneous trait, whereas SMI and Conn.D are the steadiest traits, with only 3 significantly distinct groups (Fig. 1B). Notably, the heterogeneity of females is greater than that of males for cortical traits; however, it is milder for trabecular traits (Fig. S1A,B for males and S1C-D for females).

To examine the inter-dependencies between the traits, we assessed their pairwise Pearson correlations (Table S1A). The strongest correlation was between BV/TV and Tb.N (Pearson’s *r* = 0.94), and the weakest was between Tb.N and vBMD (*r* < 0.01). There was also a moderately high correlation between Ct.Th and Tb.Th (*r* = 0.61; Table S1A). The correlation between the sexes for each trait (Table 1) ranged from *r* = 0.75 (Tb.Sp) to *r* = 0.20 (Ct.Th). Body weight (range = 17.4–35.0 g) did not significantly correlate with any of the traits (*r* = 0.01 for Conn.D to *r* = 0.19 for Ct.Th; data not shown). After males were separated from females, the correlation slightly increased, yet remained low. A weak correlation was found between weight and Tb.N, SMI, and Ct.Th for females (Pearson’s *r* = −0.20, 0.23, and 0.25, respectively), and between weight and Tb.Th and Tb.Sp (Pearson’s *r* = 0.25 and −0.25) for males (data not shown). Femur length ranged from 13.06 to 16.08 mm (mean = 14.63 mm); it correlated with weight (r = 0.43) but not with any other examined trait. That there isn’t notable association between bone length and the various femoral features indicates that the compartment is independent of bone length. Further support that the ROI is unaffected by length is provided in^25,26^.

**Table 1:** Heritability, sex correlations, and covariate interactions for trabecular and cortical traits.

Trait	H^2^	logP	H^2^n	Sex Cor.	Interactions %
BV/TV	0.61	12.43	0.87	0.669575	—
Tb.N	0.63	13.76	0.88	0.762846	—
Tb.Th	0.54	9.08	0.83	0.703989	34.70
Conn.D	0.56	9.90	0.84	0.518804	—
SMI	0.55	9.43	0.84	0.298417	26.02
Tb.Sp	0.63	13.45	0.88	0.754563	—
vBMD	0.62	12.15	0.87	0.626882	53.92
Ct.Th	0.51	7.63	0.82	0.205568	41.07

H^2^ is the broad-sense heritability (which includes epistatic and environmental influences); logP is the negative 10-base logarithm of the P-value; H^2^n is the line-mean heritability; Sex Cor. is the sex correlation of each trait; and interactions % refers to the relative contribution of the cumulative covariate-interactions, which include sex, age, batch, month, season, year, and experimenter (see Table S2).

Interestingly, whereas in classical strains such as C57BL/6J, male BV/TV is usually twice that of female, here it did not display a statistically significant sex effect. We therefore examined more closely the 15 lines with sufficient observations in both males and females (Table S1B). Of these, 5 lines had significantly higher BV/TV in males than in females, and 1 line had 118% higher BV/TV in females than in males with borderline significance (p = 0.056). The remaining 9 lines did not exhibit a statistically significant sex effect (Table S1B).

### Heritability and confounder control

We evaluated the effects of the covariates sex, age, batch, month, season, year, and experimenter on each trait. Ages ranged from 9 (n = 6) to 13 (n = 9) weeks; the mice were dissected in 20 batches over a three-year period during winter, spring, or summer, by two experimenters. µCT scanning was also performed in batches; slight fluctuations in the X-ray source might have affected the signal. Whereas age alone (between 9 and 13 weeks) had no significant effect on any trait, sex affected Ct.Th; batch affected Tb.Th, vBMD, and Ct.Th; the month affected Tb.Sp, vBMD, and Ct.Th; the season and year affected vBMD and Ct.Th; the experimenter affected Tb.Th and Ct.Th (Table S2). The cumulative effect of the covariates’ pairwise interactions was noted for Tb.Th, SMI, vBMD, and Ct.Th (Tables S2 and S3).

We then estimated the broad-sense heritability (H^2^) of each trait among the CC lines, which includes additive and non-additive epistatic effects and gene-environment interactions (Table 1). The greater H^2^ value was for Tb.N (0.63, log*P* = 13.76, where log*P* denotes the negative 10-base logarithm of the *P* value and tests the null hypothesis that the heritability is zero), and that Ct.Th had the smallest H^2^ value (0.51, log*P* = 7.63). We also calculated the heritability for the mean values in each line to obtain a better representation of the percentage of the genetic contribution to the phenotypic heterogeneity by incorporating H^2^ and the average number of lines^27^. This defines H^2^n, which is directly proportional to H^2^ (see Methods; Table 1) and ranges from 82 (Ct.Th) to 88% (Tb.N and Tb.Sp).

Overall, the cortical traits were more influenced by covariates than by trabecular traits; they were particularly sensitive to sex, batch, and season. The non-significant sex effect observed in our cohort contrasts with the well-established skeletal sexual dimorphism in common inbred mice^28^. Our current data suggest that sex has a more complex effect that is dependent on combined environmental and genetic factors, and that it is evidently cohort composition dependent, i.e., it has an element of randomness.

### Association analysis for microarchitectural traits highlights 5 QTLs

**Figure 2:**
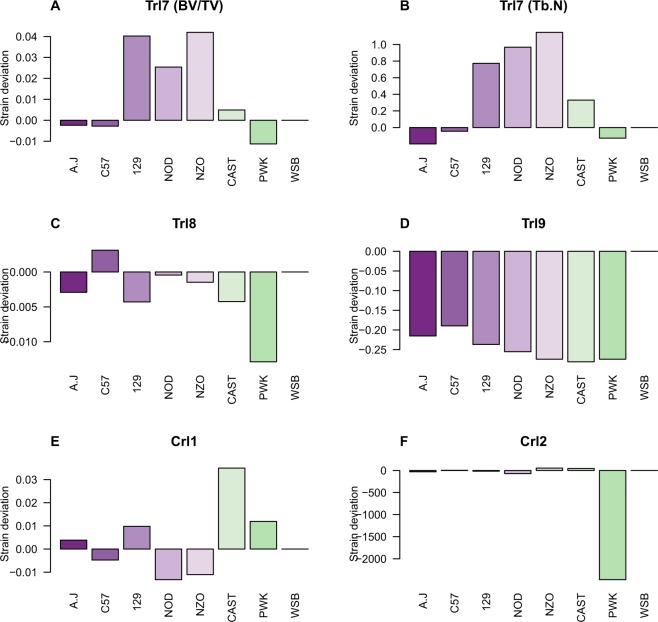
Ancestral effects relative to WSB. The y-axis represents the strain deviation relative to WSB; the x-axis represents the different strains of the eight CC founders. **(A**–**F)** *Trl7* to *Trl9*, *Crl1*, and *Crl2*, respectively.

We next measured the genetic association between each trait and the CC genomes using a haplotype-based test of association. These analyses yielded 5 distinct QTLs that were genome-wide significant at P < 10^−6^. BV/TV and Tb.N contained a marked peak at a locus of ~0.45 Mb between 116.5 and 116.9 Mb on chromosome 11, with peak log*P* values of 7.6 and 6.8. For Tb.Th, Tb.Sp, Ct.Th, and vBMD, we identified different QTLs on chromosomes 4, 5, 4, and 3, respectively. Conn.D and SMI lacked significant QTLs and were thus dismissed (Fig. S2); Conn.D displayed a borderline peak in a region that matches the peak identified for BV/TV and Tb.N. The 5 QTLs we describe are hereafter referred to as *Trl* (trabecular-related locus) 7 to 9, and *Crl* (cortical-related locus) 1 and 2 (respectively, for BV/TV and Tb.N, Tb.Th, Tb.Sp, Ct.Th, and vBMD; this is in agreement with our previous report^16^ that introduced *Trl* 1 to 6. The 95% width of the confidence intervals ranged from 6.4 to 15.6 Mb for the *Trl*s, and between 8.5 and 10.8 Mb for the *Crl*s (Table 2 and Fig. S3). We measured the contribution of each CC founder to the QTLs, relative to the wild-derived strain WSB/EiJ (Fig. 2). *Trl*7 is mostly affected by the classic laboratory strains 129S1/SvImJ, NOD/LtJ, and NZO/HiLtJ. Notably, the other traits were more strongly driven by the following wild strains: *Trl*8 and *Crl*2 by PWK/PhJ, *Trl*9 by WSB/EiJ, and *Crl*1 by CAST/Ei.

**Table 2:** Positions of QTLs associated with trabecular and cortical traits.

QTL	Trait	Chr	logP	99th % thresh.	H^2^_r_	Simulation					
						50% CI (Mb)		90% CI (Mb)		95% CI (Mb)	
						Position	Width	Position	Width	Position	Width
*Trl7*	BV/TV	11	7.60	4.90	0.69	116.6–116.7	0.12	113.6–118.1	4.50	112.1–118.3	6.40
*Trl7*	Tb.N	11	6.80	4.84	0.71	116.6–116.87	0.29	114.2–118.35	4.15	112.41–118.68	6.27
*Trl8*	Tb.Th	4	8.00	4.50	0.61	117.2–117.58.	0.32	113.05–125.54	12.49	110.87–126.52	15.65
*Trl9*	Tb.Sp	5	9.40	6.01	0.78	105.78–106.14	0.35	101.6–109.11	7.54	99.8–110.39	10.62
*Crl1*	Ct.Th	4	8.20	4.80	0.80	9.29–9.72	0.43	4.0–11.7	7.70	3.4–11.8	8.49
*Crl2*	vBMD	3	9.80	4.93	0.86	97.2–97.4	0.20	94.4–103.1	8.50	93.22–104.3	10.80

Chr = chromosome; logP = negative 10-base logarithm of P value; Sig = genome-wide significance level; 99th % threshold logP = threshold used to define cut-off for QTL peaks (Fig. 4); ${{{\rm{H}}}_{{\rm{r}}}}^{2}$ = regional heritability (the proprtion to which the locus explains the phenotypic variability). Positions and widths of the simulation-based 50, 90, and 95% CIs are given.

**Figure 3:**
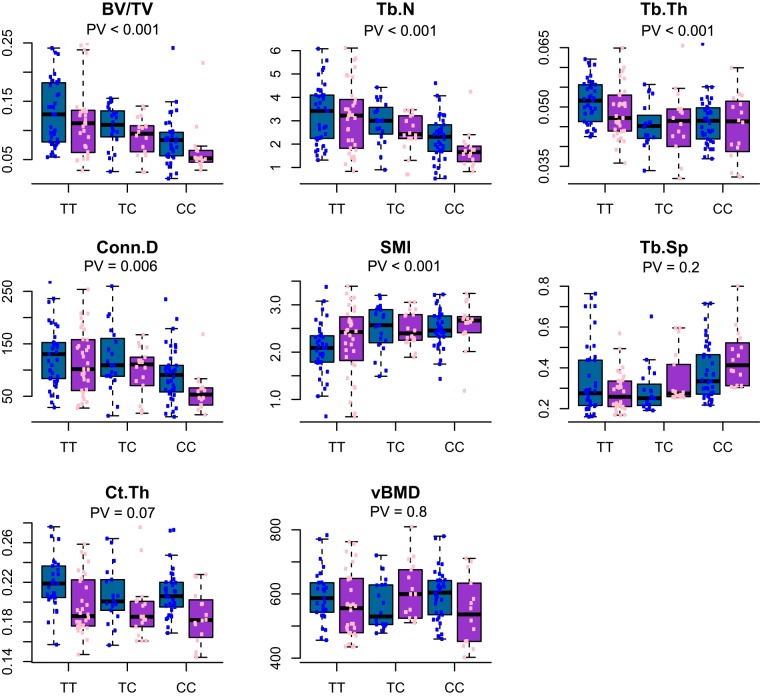
Allelic variants at *Tlr7*. Distribution of traits at the marker JAX00032223 across bearers of homozygous and heterozygous alleles, separated by sex. The x-axis is the allelic variation at the marker; the y-axis represents the trait value. PV denotes the *P*-value determined by multi-way ANOVA with the examined trait as the dependent variable and the covariates as independent variables.

At the sequence SNPs most adjacent to the peak of *Trl7*, JAX00032223 (9 bp downstream of rs247017068), we found that lines with a T:T allele mostly congregate at the higher end of the BV/TV and Tb.N values (mean BV/TV = 17%); lines with a C:C allele are at the lower end (mean BV/TV = 10%), and those with a C:T variant are at the intermediate range (Fig. 3). Generally, the more a given trait correlates with BV/TV, the more differentiated its C:C and T:T variants are, at JAX00032223. This is accentuated in vBMD, where the weak correlation with BV/TV (Table S1A) fits the leveled C:C and T:T groups (P value = 0.8 using Welch’s two sample t-test). See Table S4 for information about how the lines spread across the alleles.

### Candidate genes identified by merge analysis and RNA-seq

**Figure 4:**
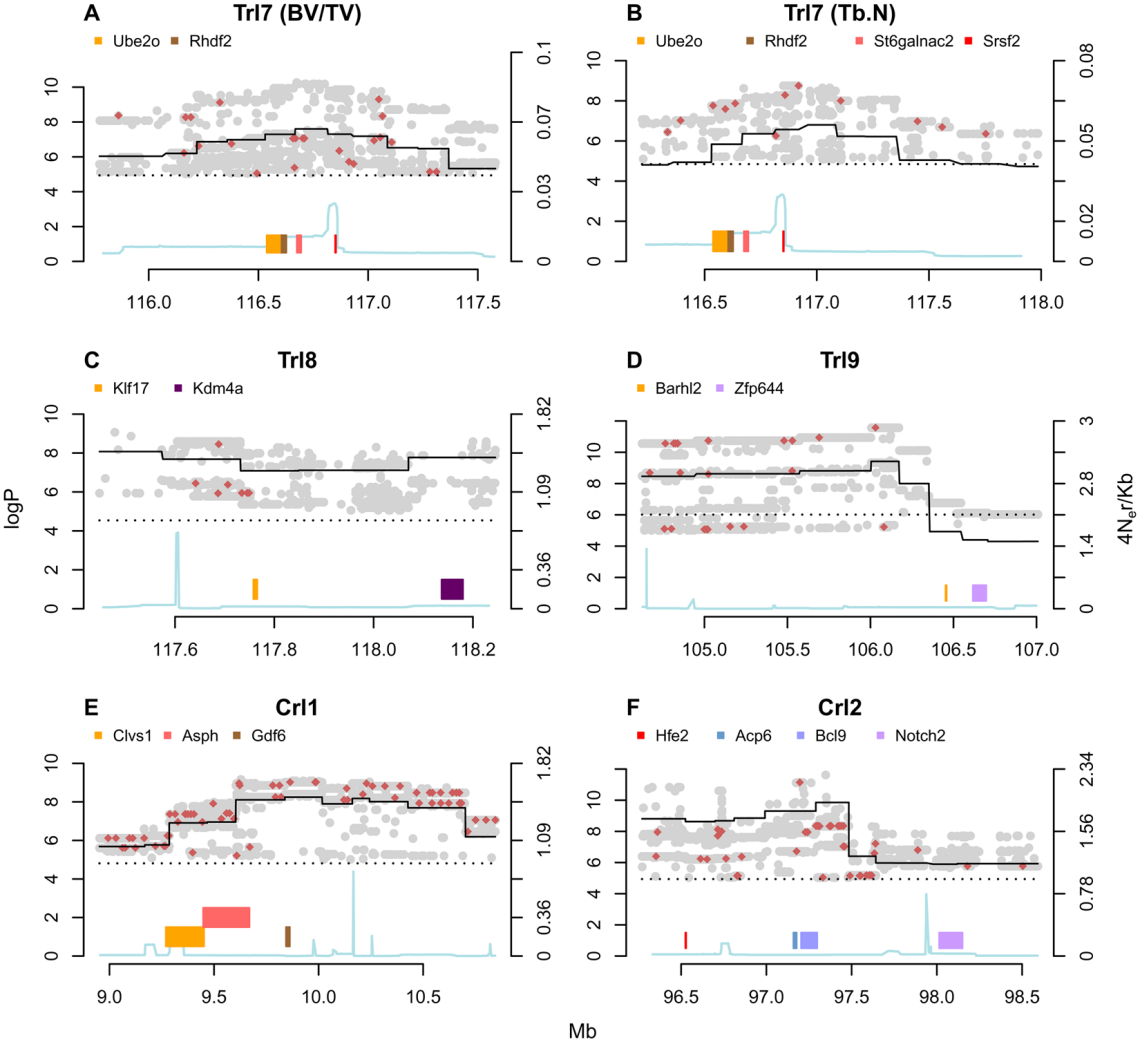
Merge analysis. The x-axis represents the position on the genome in Mb; the left y-axis represents the log*P* score; the right y-axis represents the recombination rate scale; colored bars are genes (note that only strong putative candidate genes are shown; readings below log*P* = 4 are omitted for brevity); the cyan line denotes the recombination rate; the black continuous line denotes the pre-merge test result; the dashed line denotes the 99% permutation threshold. **(A–F)** Trl7 of BV/TV, Trl7 of Tb.N, Trl8 of Tb.Th, Trl9 of Tb.Sp, Crl1 of Ct.Th, and Crl2 of vBMD, respectively.

Merge analysis uses the catalogue of variants segregating in the eight CC ancestors to impute the genotypes in the CC, given the haplotype mosaic structure of each CC line, to test the association between the imputed variant and the phenotype, and to compare the strength of association (merge log*P*) at the imputed variant with the haplotype-based test of association at the same locus^29^. Candidate causal variants, if they exist, are expected to have higher log*P* values than does the haplotype-based test. We found that *Trl*7 had the highest density of polymorphisms (gray and red dots in Fig. 4) with merge-log*P* values above the haplotype log*P*s (the continuous black line in Fig. 4), whereas *Trl*8 and *Crl*2 had very few. The merge log*P* values of the two latter loci congregated more upstream, in accordance with the left-skewness of their respective CI simulations (Figs. 4 and S3A; Table S5).

Two genes scored particularly high in the merge test: *Aanat* and *Rhbdf2* (merge strength of 15.38 and 14.07 [log*P* = ~8.5 and ~9], respectively), and are therefore probably associated with *Trl7*. Other genes such as *Ube2o*, *Cybg*, and *Sphk* were ranked in successive order in our merge analysis (Fig. 4 and Table S5). Each gene, together or separately, is a strong potential contributor to the corresponding phenotype.

**Figure 5:**
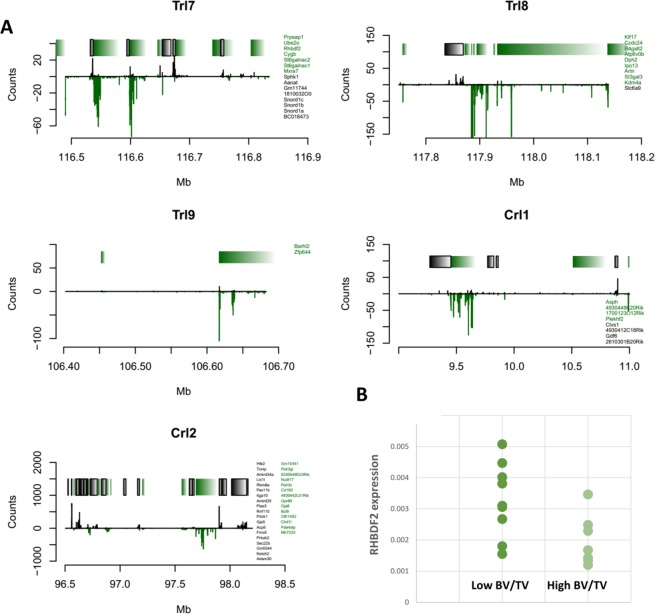
(**A**) RNA-seq of osteoclasts. Gene names are displayed on the right of each plot. Green represents plus-stranded genes; black represents minus-stranded genes. The y-axis represents the raw expression count, where the negative scale refers to the minus-stranded gene count. Each bracket corresponds to a particular gene; left-to-right green (black) brackets fit green (black) top-to-bottom gene names. (**B**) mRNA expression in the femur of male mice. mRNA expression values for *Rhbdf2* were normalized against *Gadph* from two of each extreme BV/TV group, 2 lines with low (bold) and 2 with high (light green); *Rhbdf2* expression in the low BV/TV lines was significantly higher than in the high BV/TV lines, p = 0.005, t-test, n = 9 and 7, respectively.

For the functional validation of our GWAS candidate genes, we first assessed whether our leading putative genes are expressed in bone cells. Although this criterion is widely accepted^30^, it is rather simplistic, since many extra-skeletal organs have been shown to affect bone homeostasis. Nevertheless, the majority of validated GWAS genes regulating bone mass are expressed in bone tissue^31,32^. In line with this assumption, we analyzed publicly available RNA-seq datasets of osteoclasts (Fig. 5A) and osteocytes (Fig. S4). These datasets were published as Gene Expression Omnibus [GEO] accession number GSE72846^33^ and GEO accession number GSE54784^34^. We focused on local maxima that span ~0.5 Mb in and around the peaks suggested by the merge analysis for each QTL. From the raw count reads, we found that all loci displayed gene expression differential to some degree. In *Trl*7, *Ube2o* and *Rhbdf2* were both expressed and had a comparable read count in osteocytes and osteoclasts; in the same locus, *Mxra7* had a strong presence in osteocytes (Fig. S4) but was expressed at a low degree in osteoclasts (Fig. 5A); notably, *Aanat* expression was negligible in both datasets (Figs. 4 and S4).

To further assess the gene that most likely contributes to the phenotype (BV/TV), we collected total bone RNA from mice of extreme lines for BV/TV, based on Duncan’s LSR (Fig. 1B), and measured the expression of *Aanat* and *Rhbdf2*, the 2 genes that showed the highest merge score (Table S5). We randomly selected 2 lines out of the 5 displaying the highest BV/TV values and 2 lines from the LSR group with the lowest BV/TV values. We found that although the expression of *Aanat* was undetectable after 35 cycles, *Rhbdf2* expression was higher in the 2 lines characterized by relatively lower BV/TV. For a more accurate assessment, we calculated the Pearson’s correlation coefficient between the RNA expression values and the measured BV/TV in each line and found that *Rhbdf2* exhibited a strong correlation (r = −0.92) between RNA levels and BV/TV (see Fig. 5B and Table 3).

**Table 3:** Total femoral RNA expression in extreme lines related to BV/TV.

Line	BV/TV	Aanat	Rhbdf2
IL-72	0.097	ND	1.00
IL-57	0.094	ND	0.81
AU8016	0.161	ND	0.60
OR3393	0.210	ND	0.48
Correlation with BV/TV			**−0.93**

RNA expression (corrected for Gapdh, normalized to line 72) in the femur of extreme lines: low (top two rows) and high (bottom two rows) BV/TV. Note the negative meaningful correlation of Rhbdf2 with BV/TV and the undetectable expression levels of Aanat (ND).

### Validation of the skeletal role of Rhbdf2 in knock-out mice

**Figure 6:**
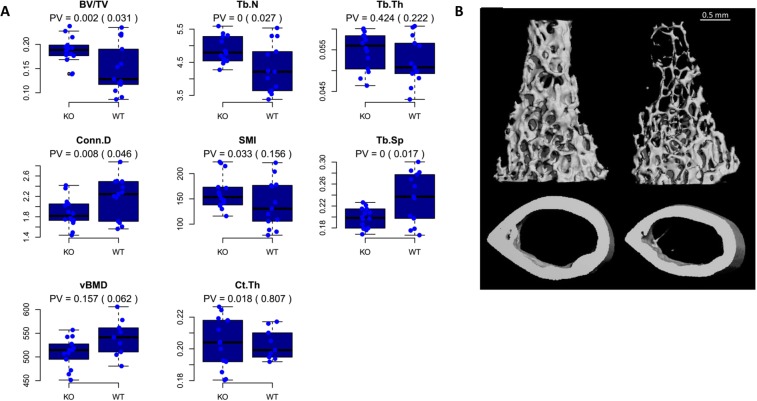
High trabecular bone mass in *Rhbdf2* knockout mice. µCT analysis in the femora of 11-week-old KO (left) versus WT (right) male mice. (**A**) Quantitative morphometric parameters presented as box-and-whisker plots, in which the horizontal black line is the median; whisker limits are minimum and maximum values; and the blue bar is the inter-quantile range. PV, *P* value KO vs WT. (**B**) Representative trabecular (top) and cortical (bottom) bone 3D reconstructions for *Rhbdf2* knockout (left) and wild-type (right) mice.

The femora of male mice (n = 14; mean length = 16.3 mm) null at *Rhbdf2* (on a C57BL/6 J background) were collected and gauged for the same morphometric traits used for the CC cohort in the exploratory section, namely, BV/TV, Tb.N, Tb.Th, Conn.D, SMI, Tb.SP, Ct.Th, and vBMD. They were compared to their wild-type (WT) counterparts (n = 13; mean length =14.62 mm. See Fig. 6).

We found that *Rhbdf2*^−/−^ mice had a significant bone phenotype; in line with our GWAS data, *Rhbdf2*^−/−^ mice displayed a highly significant increase in BV/TV and Tb.N (Fig. 6). As expected, *Rhbdf2* KO also affected other microstructural parameters, partly due to the high correlation between the trabecular traits. We also observed a significant difference between KO and WT animals regarding Tb.Sp (*P* value = 0.017) and Conn.D (*P* value = 0.046). The Tb.Th and vBMD values were not affected by the knockout (Fig. 6). Interestingly, the cortical compartment did not display a peak in the vicinity of *Trl*7 in the CC animals, and it was also not significantly affected by *Rhbdf2* KO.

## Discussion

Here we characterized several key microstructural properties of the mouse femoral bone in order to (i) assess the extent to which they are heritable, (ii) determine what environmental perturbations they are prone to, and (iii) identify candidate genes that control them. To amplify QTL calls, we used a set of 34 lines that included 20 lines already used in our previous study^16^; thus, we were able to discover the strongest QTLs in our entire cohort.

Although the heritability estimates exceeded 50% for all traits and confirmed our previous findings, here the degree to which sex explains the phenotypic variation was very subtle, and appeared only for the cortical traits. As detailed above, BV/TV was often sex unbiased, and in one strain we found that BV/TV was actually higher in females, which contrasts with the generally reported higher BV/TV in males. Notably, our mouse panel was not designed to specifically address the question of sex differences; increasing the number of replicates can aid in assessing it conclusively. Nevertheless, it should be noted that examining also the non-significant trends (<15% difference) revealed that 10 out of 15 lines agree with the generally higher BV/TV values found in males (Table S1B). Perhaps more interesting is the fact that 2 lines exhibited higher BV/TV values in females; one was statistically not significant, the other line displayed >2-fold higher values in females, with borderline statistical significance (+24.9%, p = 0.569 and +117.8%, p = 0.056). It therefore seems likely that an individual’s genetic background also influences sexual dimorphism in bone microarchitecture. Notably, the heritability of Tb.N and Tb.Th (63 and 54%, respectively, Table 1) is similar to that recently observed by Karasik *et al*.^35^ for human tibia (60 and 52%, respectively). We found a total of five QTLs in six traits: BV/TV and Tb.N shared one QTL, and Tb.Th, Tb.Sp, vBMD, and Ct.Th yielded one each. These QTLs are referred to as *Trl*7-9, and *Crl*1-2, respectively. Our merge analysis highlighted 2 putative genes under the 50% confidence interval (Table S5) because of their strong association with the BV/TV phenotype. Admittedly, even though we validated *Rhbdf2* using our KO model, *Trl7* may have been driven by other genes in this QTL, such as *Ube2O*, and/or *Cygb*. *Ube2o* (Ubiquitin Conjugating Enzyme E2 O) encodes an enzyme that is an important interactant of SMAD6. Ube2o monoubiquitinates SMAD6, and thereby facilitates the latter to bind BMP-1 receptors^36^. The signal transduction of BMP-1 is consequently impaired^37,38^, and endochondral bone formation, instead of ossification, is favored. Importantly, 4-week-old SMAD6-overexpressed mice have significantly lower humeral and vertebral BV/TV than do WT controls^37^. *Cygb* encodes cytoglobin, a heme-containing protein with peroxidase activity^39^. *Aanat* encodes a key regulator of melatonin biosynthesis, which controls light/dark cycles^40^.

*Rhbdf2* (Rhomboid 5 Homolog 2) encodes the iRhom2 protein, a polytopic membrane protein that is a catalytically inactive member of the rhomboid intramembrane serine protease superfamily^41^. iRhom2 is necessary in macrophages for the maturation and release of the inflammatory cytokine tumor necrosis factor α (TNFα): it acts in the trafficking of TACE, the protease that releases active TNFα from its membrane-tethered precursor^42,43^. iRhom2 is also implicated in the signaling of EGF-family growth factor^44–46^. According to a recent report on its role in the trafficking of another protein, STING, it appears that iRhom2 plays a wider role in regulating membrane trafficking^47^. iRhom2 was also implicated in the regulation of CSF1R (macrophage stimulating factor 1 receptor), a critical regulator of osteoclast differentiation and survival^43,48–50^. *In vivo*, *Rhbdf2* has been implicated in esophageal cancer, wound healing, bone marrow repopulation by monocytic cells, and inflammatory arthritis^45,51–53^.

We observed a differential expression between osteocytes and osteoblasts, which hints at their involvement in a cell-specific mechanism underlying most genes except *Aanat* in *Trl*7. This was mirrored by a mRNA-expression assay in which *Aanat* was undetectable (Table 3). On the other hand, *Rhbdf2* expression in bone was confirmed by the analyzed GEO RNA-seq data (Figs. 5 and S4) and our total femoral RNA analysis. We also found that *Rhbdf2* gene expression in bone and BV/TV was tightly correlated in the extreme CC lines (Table 3). To validate our finding, we compared the bone phenotype of *Rhbdf2*^−/−^ with that of wild-type controls. Strikingly, *Rhbdf2* deletion affected all the examined trabecular traits. Although the effects on BV/TV and Tb.N were in line with the genetic mapping, the *Rhbdf2* locus did not appear in any of the other traits. An obvious explanation would be the difference between polymorphisms segregating in the CC and deletion of KO. Indeed, Tb.Sp differed greatly when comparing the *Rhbdf2*^−/−^ and control mice, although it did not appear in the initial mapping. This might be due to (i) the great diversity of the wild-type mice in Tb.Sp, (ii) the need for a complete knockout rather than only an SNP to detect significant changes in Tb.Sp, (iii) the SNPs give rise to *Trl*7, which are functioning variants, with differential behavior affecting only BV/TV and Tb.N. The significant QTL peak we found in this GWAS for BV/TV and Tb.N revealed a gene that has a crucial skeletal function in the trabecular bone compartment. Importantly, there are no coding polymorphisms in *Rhbdf2* annotated to cause severe gene disruption; therefore, it is likely that a regulatory polymorphism is causal.

Further work is needed to identify the mechanism by which iRhom2 controls bone homeostasis; one possible direction could involve a positive feedback mechanism that leads to the differentiation of macrophages to osteoclasts. Indeed, iRhom2 stimulates the secretion of TNFα by macrophages^50,54^; it hyperactivates EGFR^45,55^ and regulates CSF1R^52,56^. Although *Rhbdf2* is expressed in both the osteocyte and osteoclast lineages, one cannot rule out the possibility that if this gene regulates bone remodeling, it may do so by virtue of its expression in non-skeletal cells. We note that support of the effect of *Rhbdf2* on the femoral features outlined here does not conclude that its mechanism relates directly either to reduced bone formation or suppression of osteoclast activity, as further cell-based assays might determine.

In summary, we have identified several putative genes, some of which are newly linked to bone biology. A confirmation of one such gene, *Rhbdf2*, provides conclusive evidence of its effects on bone microstructure and in general, this constitutes the first demonstration of a physiological role for *Rhbdf2*. This finding will most likely assist to identify the mechanism underlying the action of *Rhbdf2*, and its possible contribution to bone loss and bone fractures in humans.

## Materials and Methods

### Mice

Mice aged 9 to 13 weeks (male *n* = 103; female *n* = 71), from 34 different CC lines (having an average of 5 mice per line) were used in this study. The mice were at inbreeding generations of 11 to 37, which correspond to a genetic homozygosity of 80 to 90%, respectively. They were bred and maintained in the small animal facility at the Sackler Faculty of Medicine, Tel Aviv University (TAU), Israel. See^16^ for details. All experimental protocols were approved by the Institutional Animal Care and Use Committee (IACUC M-13-014) at TAU, which follows the NIH/USA animal care and use protocols. The *Rhbdf2* knock-out mice (having a C57BL/6J background) and their WT littermates were obtained from a colony maintained at the University of Oxford, approved by license PPL80/2584 of the UK Home Office.

### Specimen collection and preparation

Mice were euthanized with 5% Isoflurane inhalation. Cervical dislocation was performed approximately one minute after breathing stopped. Left femora were harvested and fixed for 24 hours in 4% paraformaldehyde solution, and then stored in 70% ethanol. *Rhbdf2* knock-out model mice were prepared as in^49^. We selected only male *Rhbdf2* mice to avoid menstrual cycle effects.

### μCT evaluation

Whole left femora from each mouse were examined as described previously^16,57^ by a μCT system (μCT 50, Scanco Medical AG, Switzerland). Whole femora were scanned at a 10 µm isotropic resolution, 70 kVp energy, 200 µAmp intensity, 1200 msec integration time, and with 1000 projections. The mineralized tissues were differentially segmented by a global thresholding procedure; we used a different threshold for the cortical (224 permil) and trabecular (160 permil) bone compartments. The signal of our Xray tube is routinely checked every 1–2 weeks using the calibration phantom provided by the manufacturer and recalibration is performed when the signal fluctuates by more than 2.5%. During this study, the scanner was recalibrated twice. For a detailed analysis of trabecular bone, we defined a region of interest of 3 mm height ending distally at the proximal tip of the primary spongiosa (=Total Volume, TV). Morphometric parameters included trabecular bone volume fraction (BV/TV; %), trabecular thickness (Tb.Th; µm), trabecular number (Tb.N; mm^−1^), trabecular connectivity density (Conn.D; mm^−3^), the trabecular structure model index (SMI), and trabecular separation (Tb.Sp; mm). Cortical bone analysis was performed in a 1 mm-height ring at the midshaft. It consisted of volumetric bone mineral density (vBMD; mgHA/cm^3^ [mg Hydroxy-Apatite per cm^3^]) and cortical thickness (Ct.Th; mm). vBMD was computed by averaging the mineral content inside the periosteal envelope of the cortical ring, including the bone marrow. All parameters were generated and termed according to the guidelines for assessment of bone microstructure in rodents using micro–computed tomography^58^.

### Genotyping

A representative male mouse from each line was initially genotyped with a high mouse diversity array (MDA), which consists of 620,000 SNPs (Durrant *et al*., 2011). After about two intervals of 4 generations of inbreeding, all the CC lines were re-genotyped by a mouse universal genotype array (MUGA, 7,500 markers), and finally with the MegaMuga (77,800 markers) SNP array to confirm and enrich their genotype status^19^. For details, see^16,22^.

### Statistical analyses and data acquisition

All statistical analyses were performed with the statistical software R (R core development team 2009), including the package happy.hbrem developed by Mott *et al*.^59^. False discovery rate (FDR) was calculated using the p.adjust function in R, with the method “BH” (Benjamini-Hochberg^60^), keeping the FDR less than or equal to 0.01.

#### Heritability and covariate effects

Broad-sense heritability (H^2^) was obtained for each trait by fitting the trait (the independent variable) to the CC line label in a linear regression model that incorporates relevant covariates (sex, age, batch, month, season, year, and experimenter). For details, see^16^. H^2^_n_ (line-mean heritability) was derived from H^2^ as in Atamni *et al*.^27^.

Regional heritability (H_r_^2^) was hereafter computed by ANOVA as in the broad-sense heritability computation, except here a null linear regression fit was compared with a genetic linear regression fit with the probability matrix of the founder descent at the peak QTL as the explanatory variable.

#### Confidence intervals

Confidence intervals (CIs) were obtained by simulations in which residuals of the original linear regression fit at the peak of each QTL were resampled, to follow the data generating process; 100 random intervals of varying lengths within 7–10 Mb of the original loci were then rescanned to determine the strongest *P*-value. As in Durrant *et al*.^22^, 1000 QTLs were simulated. See^16,22^ for details.

#### RNA-seq data

RNA-seq data from osteoclasts and osteocytes were obtained from the gene expression omnibus (GEO) database (accession numbers GSE72846 and GSE54784) and mapped to the *mus musculus* assembly mm10 using tophat v. 2^61^. Read counts were assigned to the loci of interest using the R (R Core Team 2015) package. Genomic alignments and raw read counts were taken. For the osteocytes, the data of basal level day 3 were averaged. The R code for the above procedures will be made available upon request.

### Total femoral mRNA expression

Femora from male mice were homogenized and RNA was purified using TRIzol reagent (Invitrogen) and phenol/chloroform according to the manufacturer’s instructions^57^. An aliquot containing 1 µg of RNA was then reverse-transcribed using oligo (dT) primers and the High Capacity cDNA Reverse Transcription Kit (Invitrogen, Grand Island, NY, USA). The resulting cDNA samples were processed for real-time RT-qPCR analysis on a StepOne Real-Time PCR machine (Applied Biosystems, Grand Island, NY, USA). RNA gene expression was normalized to *Gapdh* transcript levels for each sample. We used validated primers (primerBank website https://pga.mgh.harvard.edu/primerbank/)^60,62^ for PCR as follows (F, forward; R, reverse): Aanat, (F) TGAGCGGGAAGCCTTTATCTC, (R) CTCCTGAGTAAGTCTCTCCTTGT; Rhbdf2 (F) TCACCTTGCTGGTGATCTGCAC, (R) GCCAATCCAGAAGTTCTCCTGC; Gapdh (F) ACCCAGAAGACTGTGGATGG, (R) CACATTGGGGGTAGGAACAC.

## Supplementary information

Supplementary Information

## References

[1] Wright NC (2014). The Recent Prevalence of Osteoporosis and Low Bone Mass in the United States Based on Bone Mineral Density at the Femoral Neck or Lumbar Spine. J. Bone Miner. Res..

[2] Burge R (2007). Incidence and economic burden of osteoporosis-related fractures in the United States, 2005–2025. J. Bone Miner. Res..

[3] Dhanwal DK, Dennison EM, Harvey NC, Cooper C (2011). Epidemiology of hip fracture: Worldwide geographic variation. Indian J. Orthop..

[4] Center JR, Nguyen TV, Schneider D, Sambrook PN, Eisman JA (1999). Mortality after all major types of osteoporotic fracture in men and women: an observational study. Lancet (London, England).

[5] Mizuguchi T (2004). LRP5, low-density-lipoprotein-receptor-related protein 5, is a determinant for bone mineral density. J. Hum. Genet..

[6] Richards JB (2008). Bone mineral density, osteoporosis, and osteoporotic fractures: a genome-wide association study. Lancet.

[7] Styrkarsdottir U (2008). Multiple genetic loci for bone mineral density and fractures. N. Engl. J. Med..

[8] Trikalinos TA, Salanti G, Zintzaras E, Ioannidis JPA (2008). Meta-analysis methods. Adv. Genet..

[9] Estrada K (2012). Genome-wide meta-analysis identifies 56 bone mineral density loci and reveals 14 loci associated with risk of fracture. Nat. Genet..

[10] Paternoster L (2010). Genome-wide association meta-analysis of cortical bone mineral density unravels allelic heterogeneity at the RANKL locus and potential pleiotropic effects on bone. Plos Genet..

[11] Paternoster L (2013). Genetic determinants of trabecular and cortical volumetric bone mineral densities and bone microstructure. Plos Genet..

[12] Jovanovich A (2013). Fibroblast growth factor 23, bone mineral density, and risk of hip fracture among older adults: the cardiovascular health study. J. Clin. Endocrinol. Metab..

[13] Hsu Y-H, Kiel DP (2012). Clinical review: Genome-wide association studies of skeletal phenotypes: what we have learned and where we are headed. J. Clin. Endocrinol. Metab..

[14] Styrkarsdottir U (2013). Nonsense mutation in the LGR4 gene is associated with several human diseases and other traits. Nature.

[15] Iraqi FA (2014). Heritability and coefficient of genetic variation analyses of phenotypic traits provide strong basis for high-resolution QTL mapping in the Collaborative Cross mouse genetic reference population. Mamm. Genome.

[16] Levy R, Mott RF, Iraqi FA, Gabet Y (2015). Collaborative cross mice in a genetic association study reveal new candidate genes for bone microarchitecture. BMC Genomics.

[17] Threadgill DW, Hunter KW, Williams RW (2002). Genetic dissection of complex and quantitative traits: from fantasy to reality via a community effort. Mamm. Genome.

[18] Churchill GA (2004). The Collaborative Cross, a community resource for the genetic analysis of complex traits. Nat. Genet..

[19] Collaborative Cross Consortium (2012). The genome architecture of the Collaborative Cross mouse genetic reference population. Genetics.

[20] Keane TM (2011). Mouse genomic variation and its effect on phenotypes and gene regulation. Nature.

[21] Roberts A, Pardo-Manuel de Villena F, Wang W, McMillan L, Threadgill DW (2007). The polymorphism architecture of mouse genetic resources elucidated using genome-wide resequencing data: implications for QTL discovery and systems genetics. Mamm. Genome.

[22] Durrant Caroline, Tayem Hanna, Yalcin Binnaz, Cleak James, Goodstadt Leo, Pardo-Manuel de Villena Fernando, Mott Richard, Iraqi Fuad A. (2011). Collaborative Cross mice and their power to map host susceptibility toAspergillus fumigatusinfection. Genome Research.

[23] Palmer C, Pe’er I (2017). Statistical correction of the Winner’s Curse explains replication variability in quantitative trait genome-wide association studies. Plos Genet..

[24] Keele Gregory R., Crouse Wesley L., Kelada Samir N. P., Valdar William (2019). Determinants of QTL Mapping Power in the Realized Collaborative Cross. G3: Genes|Genomes|Genetics.

[25] Bajayo A (2005). Central IL-1 receptor signaling regulates bone growth and mass. Proc. Natl. Acad. Sci. USA.

[26] Tam, J. *et al*. Involvement of Neuronal Cannabinoid Receptor CB1 in Regulation of Bone Mass and Bone Remodeling. **70**, 786–792 (2006). 10.1124/mol.106.026435 16772520

[27] Atamni HJA-T, Mott R, Soller M, Iraqi FA (2016). High-fat-diet induced development of increased fasting glucose levels and impaired response to intraperitoneal glucose challenge in the collaborative cross mouse genetic reference population. BMC Genet..

[28] Bab Itai, Hajbi-Yonissi Carmit, Gabet Yankel, Müller Ralph (2007). Micro-Tomographic Atlas of the Mouse Skeleton.

[29] Yalcin B, Flint J, Mott R (2005). Using progenitor strain information to identify quantitative trait nucleotides in outbred mice. Genetics.

[30] Grotz AK, Gloyn AL, Thomsen SK (2017). Prioritising Causal Genes at Type 2 Diabetes Risk Loci. Curr. Diab. Rep..

[31] Kemp JP (2017). Identification of 153 new loci associated with heel bone mineral density and functional involvement of GPC6 in osteoporosis. Nat. Genet..

[32] Morris JA (2019). An atlas of genetic influences on osteoporosis in humans and mice. Nat. Genet..

[33] Kim K (2016). MMP-9 facilitates selective proteolysis of the histone H3 tail at genes necessary for proficient osteoclastogenesis. Genes Dev..

[34] St. John HC (2014). The Osteoblast to Osteocyte Transition: Epigenetic Changes and Response to the Vitamin D_3_ Hormone. Mol. Endocrinol..

[35] Karasik David, Demissie Serkalem, Zhou Yanhua, Lu Darlene, Broe Kerry E, Bouxsein Mary L, Cupples L Adrienne, Kiel Douglas P (2016). Heritability and Genetic Correlations for Bone Microarchitecture: The Framingham Study Families. Journal of Bone and Mineral Research.

[36] Zhang X (2013). Fine-tuning BMP7 signalling in adipogenesis by UBE2O/E2-230K-mediated monoubiquitination of SMAD6. EMBO J..

[37] Horiki M (2004). Smad6/Smurf1 overexpression in cartilage delays chondrocyte hypertrophy and causes dwarfism with osteopenia. J. Cell Biol..

[38] Estrada KD, Retting KN, Chin AM, Lyons KM (2011). Smad6 is essential to limit BMP signaling during cartilage development. J. Bone Miner. Res..

[39] Lv Y, Wang Q, Diao Y, Xu R (2008). Cytoglobin:A Novel Potential Gene Medicine for Fibrosis and Cancer Therapy. Curr. Gene Ther..

[40] Mukherjee S, Maitra SK (2015). Gut Melatonin in Vertebrates: Chronobiology and Physiology. Front. Endocrinol. (Lausanne)..

[41] Lemberg MK, Freeman M (2007). Functional and evolutionary implications of enhanced genomic analysis of rhomboid intramembrane proteases. Genome Res..

[42] Adrain, C. *et al*. Supporting Online Material for. **225** (2012).

[43] McIlwain DR (2012). iRhom2 Regulation of TACE Controls TNF-Mediated Protection Against Listeria and Responses to LPS. Science (80-.)..

[44] Siggs O. M., Grieve A., Xu H., Bambrough P., Christova Y., Freeman M. (2014). Genetic interaction implicates iRhom2 in the regulation of EGF receptor signalling in mice. Biology Open.

[45] Hosur V (2014). Rhbdf2 mutations increase its protein stability and drive EGFR hyperactivation through enhanced secretion of amphiregulin. Proc. Natl. Acad. Sci..

[46] Li X (2015). iRhoms 1 and 2 are essential upstream regulators of ADAM17-dependent EGFR signaling. Proc. Natl. Acad. Sci. USA.

[47] Luo W-W (2016). iRhom2 is essential for innate immunity to DNA viruses by mediating trafficking and stability of the adaptor STING. Nat. Immunol..

[48] Siggs OM (2012). iRhom2 is required for the secretion of mouse TNFα. Blood.

[49] Adrain C, Zettl M, Christova Y, Taylor N, Freeman M (2012). Tumor Necrosis Factor Signaling Requires iRhom2 to Promote Trafficking and Activation of TACE. Science (80-.)..

[50] Udagawa N (1990). Origin of osteoclasts: mature monocytes and macrophages are capable of differentiating into osteoclasts under a suitable microenvironment prepared by bone marrow-derived stromal cells. Proc. Natl. Acad. Sci. USA.

[51] Issuree PDA (2013). iRHOM2 is a critical pathogenic mediator of inflammatory arthritis. J. Clin. Invest..

[52] Qing X (2016). iRhom2 regulates CSF1R cell surface expression and non-steady state myelopoiesis in mice. Eur. J. Immunol..

[53] Blaydon DC (2012). RHBDF2 mutations are associated with tylosis, a familial esophageal cancer syndrome. Am. J. Hum. Genet..

[54] Kobayashi K (2000). Tumor necrosis factor alpha stimulates osteoclast differentiation by a mechanism independent of the ODF/RANKL-RANK interaction. J. Exp. Med..

[55] Yi T (2008). Epidermal growth factor receptor regulates osteoclast differentiation and survival through cross-talking with RANK signaling. J. Cell. Physiol..

[56] Hung JY (2014). Colony-stimulating factor 1 potentiates lung cancer bone metastasis. Lab. Investig..

[57] Hiram-Bab S (2015). Erythropoietin directly stimulates osteoclast precursors and induces bone loss. FASEB J..

[58] Bouxsein ML (2010). Guidelines for assessment of bone microstructure in rodents using micro-computed tomography. J. Bone Miner. Res..

[59] Mott R, Talbot CJ, Turri MG, Collins AC, Flint J (2000). A method for fine mapping quantitative trait loci in outbred animal stocks. Proc. Natl. Acad. Sci. USA.

[60] Hochberg, Y. Controlling the False Discovery Rate: A Practical and Powerful Approach to Multiple Testing Author (s): Yoav Benjamini and Yosef Hochberg Source: Journal of the Royal Statistical Society. Series B (Methodological), Vol. 57, No. 1 (1995), Publi. **57**, 289–300 (2016).

[61] Trapnell C, Pachter L, Salzberg SL (2009). TopHat: discovering splice junctions with RNA-Seq. Bioinformatics.

[62] Spandidos A, Wang X, Wang H, Seed B (2010). PrimerBank: a resource of human and mouse PCR primer pairs for gene expression detection and quantification. Nucleic Acids Res..

