# Supplementary material for: A genome-wide association study in mice reveals a role for Rhbdf2 in skeletal homeostasis
Source: Sci Rep. 2020 Feb 24;10:3286. doi: 10.1038/s41598-020-60146-8 (PMC7039944; doi:10.1038/s41598-020-60146-8)
Supplement: Supplementary file 1 — Supplementary Information [file 41598_2020_60146_MOESM1_ESM.pdf]

A genome-wide association study in mice reveals a role for *Rhbdf2* in skeletal homeostasis

Roei Levy[1], Clemence Levet[3], Keren Cohen[1], Matthew Freeman[3], Richard Mott[4], Fuad Iraqi[2], and Yankel Gabet[1]

[1] Department of Anatomy and Anthropology and [2] Department of Clinical Microbiology and Immunology, Sackler Faculty of Medicine, Tel Aviv University, Tel Aviv 69978, Israel. [3] Dunn School of Pathology, South Parks Road, Oxford OX1 3RE, UK. [4] UCL Genetics Institute, University College London, Gower St., London, WC1E 6BT, UK.

Supplementary Material

A

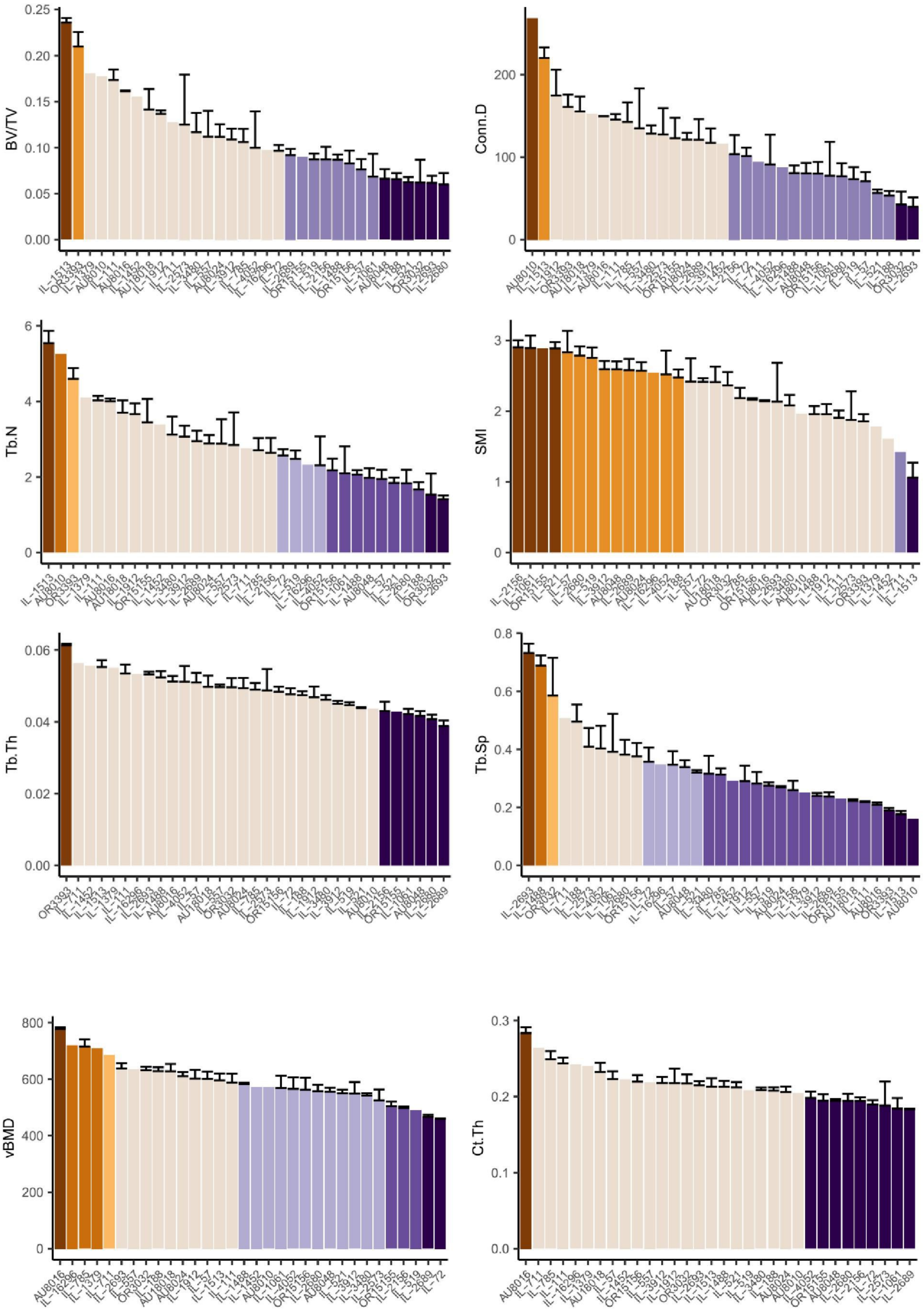

B

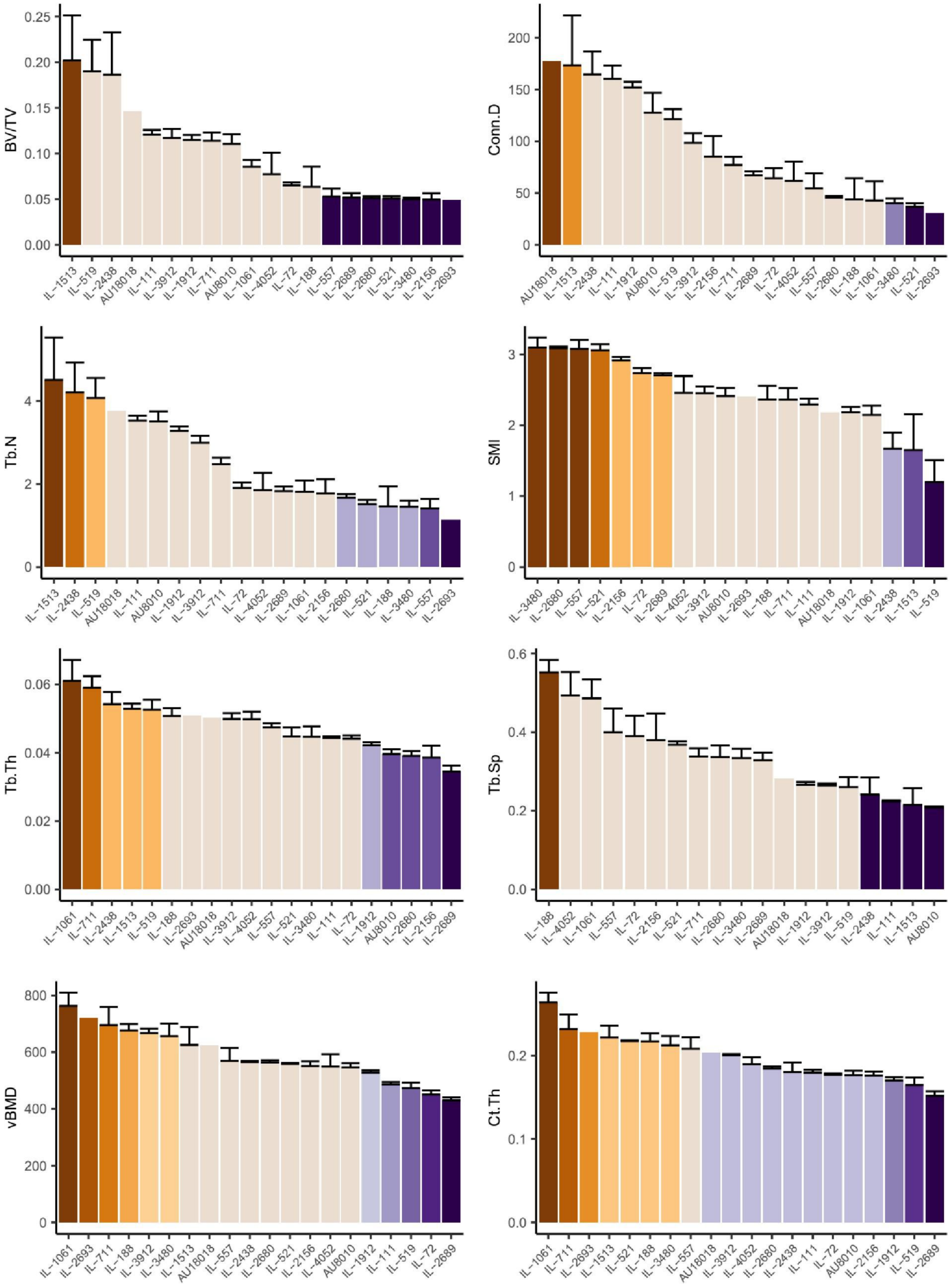
BV/TV
0.00
0.05
0.10
0.15
0.20
0.25
IL-1513 IL-519 IL-2438 AU18018 IL-111 IL-3912 IL-1912 IL-711 AU8010 IL-1061 IL-4052 IL-72 IL-188 IL-557 IL-2689 IL-2680 IL-521 IL-3480 IL-2156 IL-2693
Conn.D
0
50
100
150
200
AU18018 IL-1513 IL-2438 IL-111 IL-1912 AU8010 IL-519 IL-3912 IL-2156 IL-711 IL-2689 IL-72 IL-4052 IL-557 IL-2680 IL-188 IL-1061 IL-3480 IL-521 IL-2693
Tb.N
0
2
4
IL-1513 IL-2438 IL-519 AU18018 IL-111 AU8010 IL-1912 IL-3912 IL-711 IL-72 IL-4052 IL-2689 IL-1061 IL-2156 IL-2680 IL-521 IL-188 IL-3480 IL-557 IL-2693
SMI
0
1
2
3
IL-3480 IL-2680 IL-557 IL-521 IL-2156 IL-72 IL-2689 IL-4052 IL-3912 AU8010 IL-2693 IL-188 IL-711 IL-111 AU18018 IL-1912 IL-1061 IL-2438 IL-1513 IL-519
Tb.Th
0.00
0.02
0.04
0.06
IL-1061 IL-711 IL-2438 IL-1513 IL-519 IL-188 IL-2693 AU18018 IL-3912 IL-4052 IL-557 IL-521 IL-3480 IL-111 IL-72 IL-1912 AU8010 IL-2680 IL-2156 IL-2689
Tb.Sp
0.0
0.2
0.4
0.6
IL-188 IL-4052 IL-1061 IL-557 IL-72 IL-2156 IL-521 IL-711 IL-2680 IL-3480 IL-2689 AU18018 IL-1912 IL-3912 IL-519 IL-2438 IL-111 IL-1513 AU8010
vBMD
0
200
400
600
800
IL-1061 IL-2693 IL-711 IL-188 IL-3912 IL-3480 IL-1513 AU18018 IL-557 IL-2438 IL-2680 IL-521 IL-2156 IL-4052 AU8010 IL-1912 IL-111 IL-519 IL-72 IL-2689
Ct.Th
0.0
0.1
0.2
IL-1061 IL-711 IL-2693 IL-1513 IL-521 IL-188 IL-3480 IL-557 AU18018 IL-3912 IL-4052 IL-2680 IL-2438 IL-111 IL-72 AU8010 IL-2156 IL-1912 IL-519 IL-2689

Figure S1. Trait distribution for males and females. **(A)** Trabecular and cortical bone parameters, respectively, in male mice. **(B)** Trabecular and cortical bone parameters, respectively, in female mice. The x-axis represents the CC lines; the y-axis represents the trait means. Trabecular traits include BV/TV (%), Tb.N (mm^{-1}), Tb.Th (μm), Conn.D (mm^{-3}), SMI, and Tb.Sp (mm). Cortical traits include vBMD ($mgHA/cm^3$) and Ct.Th (mm). Color codes denote group lines that significantly differ from other groups. Lines are ordered inconsistently among the traits, according to trait-specific descending order.

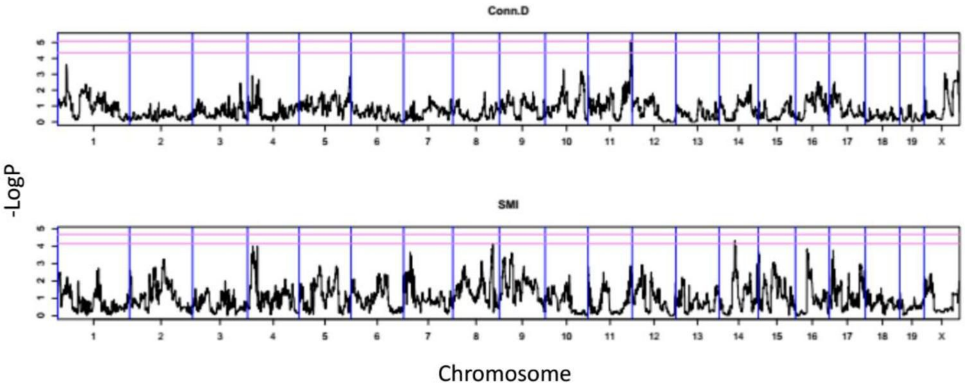

Figure S2. Haplotype association maps for trabecular Conn.D and SMI. The x-axis represents the position on the chromosome; the y-axis is the –logP value of the association. The lower threshold represents the 95th percentile of 200 simulations, and the top represents the 9th percentile. The loci above the 99% cut-off were further investigated.

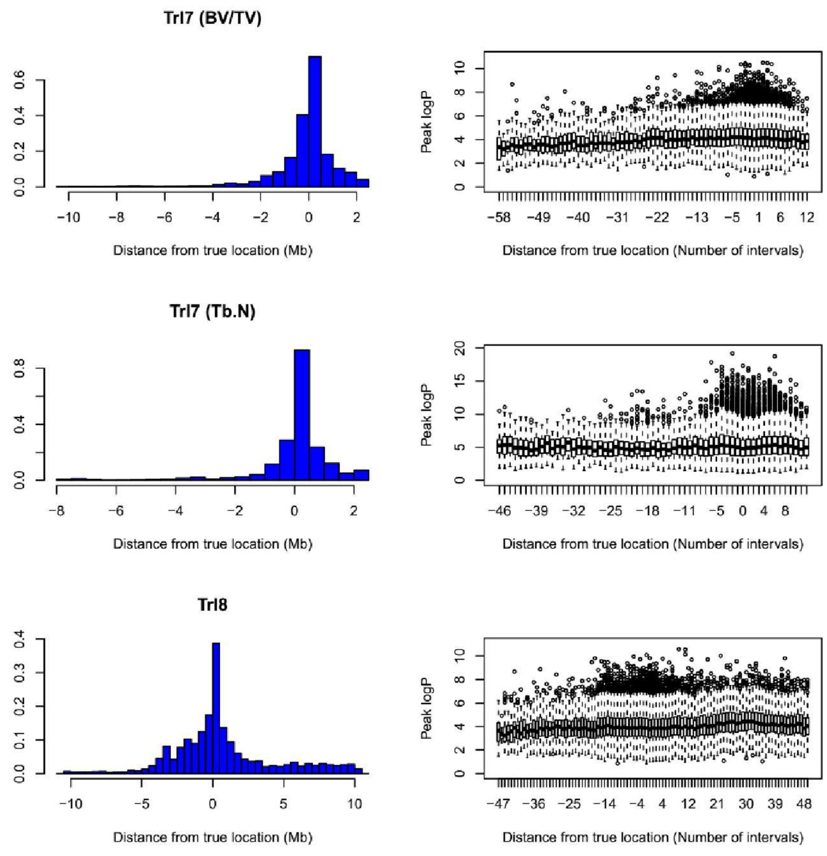
Trl7 (BV/TV)
Distance from true location (Mb)
Peak logP
Distance from true location (Number of intervals)
Trl7 (Tb.N)
Distance from true location (Mb)
Peak logP
Distance from true location (Number of intervals)
Trl8
Distance from true location (Mb)
Peak logP
Distance from true location (Number of intervals)

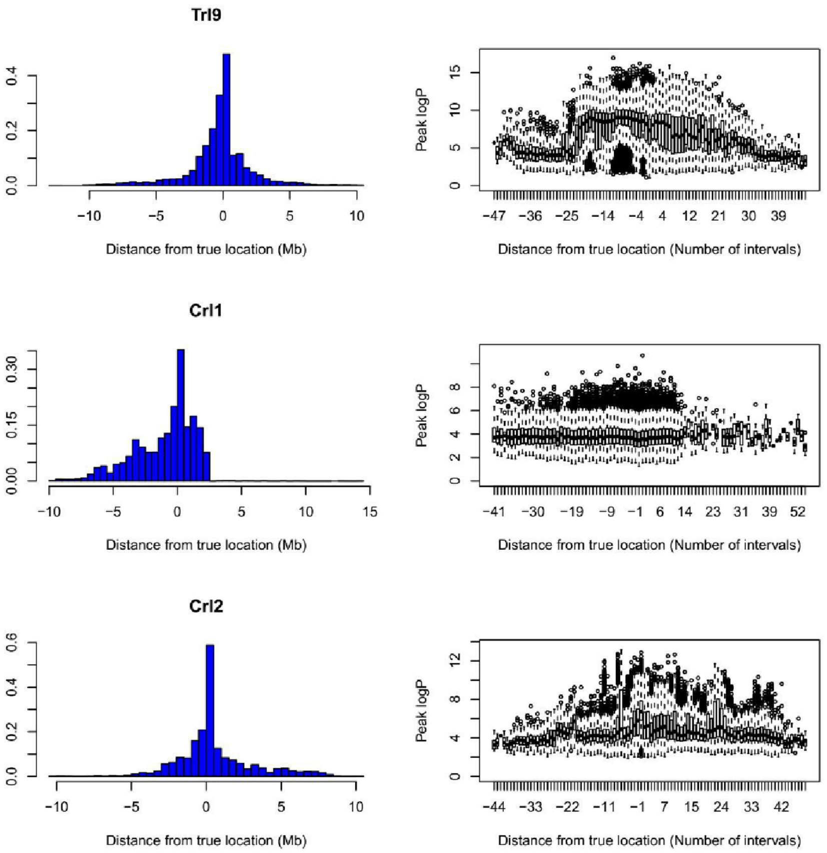
Trl9
Distance from true location (Mb)
Peak logP
Distance from true location (Number of intervals)
Crl1
Distance from true location (Mb)
Peak logP
Distance from true location (Number of intervals)
Crl2
Distance from true location (Mb)
Peak logP
Distance from true location (Number of intervals)

Figure S3. Confidence interval simulations. Loci at a neighborhood of 3-5 Mb around the original locus were simulated by permuting the residual sum of the squares of the related phenotype. The maximum logP was obtained along with its position in Mb relative to the original QTL (histograms, left panels), and with the number of markers from the original QTL (boxplots, right panels). (**A**) and (**B**) show simulation results for the BV/TV and Tb.N loci. They determined with high confidence that the peak QTL is responsible for the effect seen in the haplotype scan; thus, the narrow CI. (**C**) to (**F**) show simulation results for Tb.Th, Tb.Sp, Ct.Th, and vBMD, respectively; note the narrow CI for *Crl2* (vBMD), wide for *Trl8* (Tb.Th), and even wider for *Crl1*(Ct.Th).

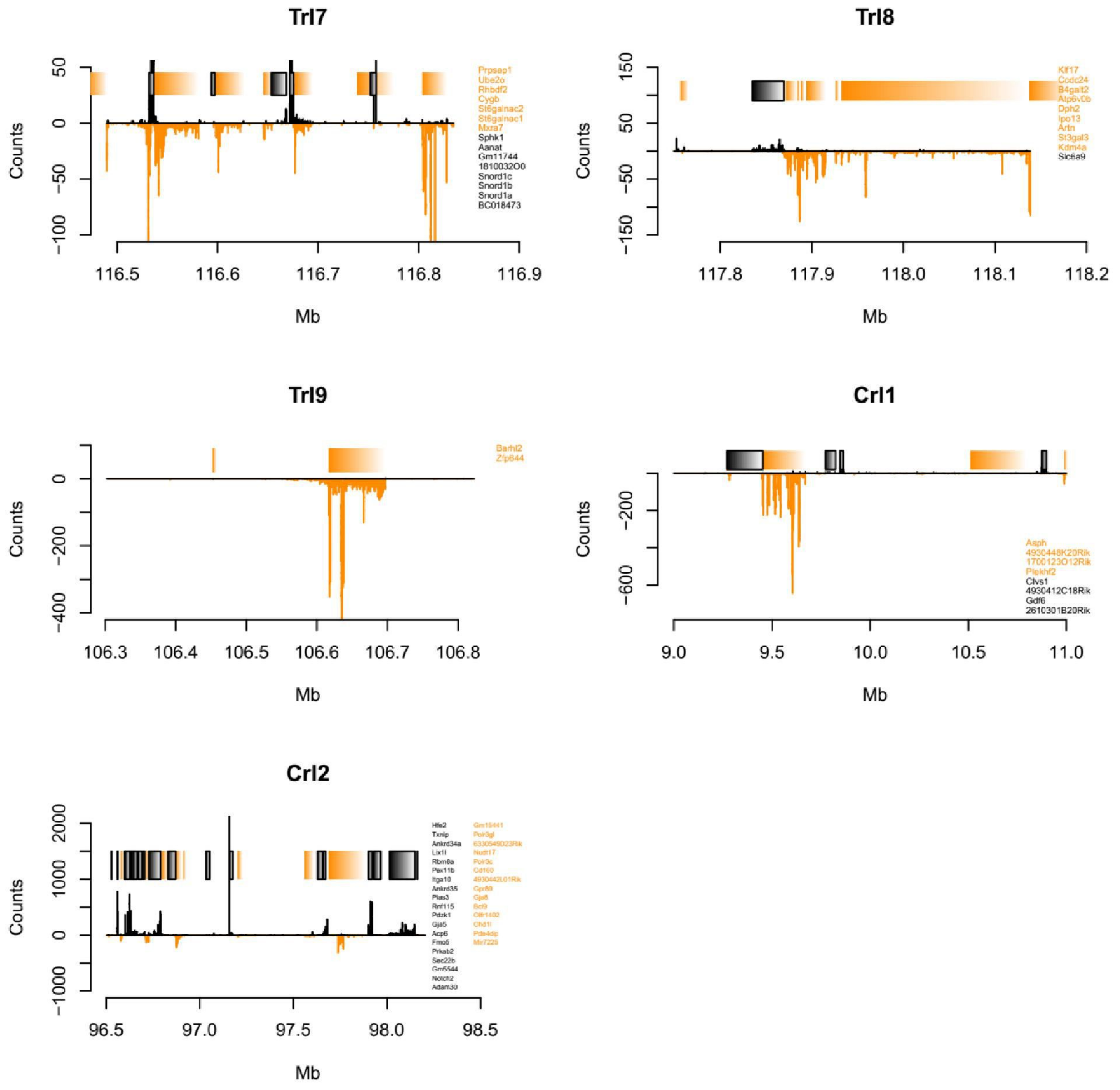

Figure S4. RNA-seq of osteocytes. Gene names appear on the right of each plot. Orange represents plus-stranded genes; black represents minus-stranded genes. The y-axis represents the raw expression count, where the negative scale refers to the minus-stranded gene count. Each bracket corresponds to a particular gene; left-to-right orange (black) brackets fit orange (black) top-to-bottom gene names.

Table S1 A. Pearson correlation coefficient between the analyzed traits

	BVTV	Conn.D	Tb.Th	Tb.N	SMI	Sp	vBMD	Ct.Th
BVTV	1							
Conn.D	**0.82**	1						
Tb.Th	0.51	0.1	1					
Tb.N	**0.94**	**0.92**	0.26	1				
SMI	**-0.83**	-0.61	-0.58	-0.71	1			
Sp	-0.56	-0.68	0.14	**-0.72**	0.13	1		
vBMD	0.11	-0.01	0.49	0.006	-0.14	0.12	1	
Ct.Th	0.24	0.06	0.61	0.12	-0.23	0.03	**0.76**	1

Correlation matrix between traits is presented as Pearson's *R*

Table S1 B. Sex effect in BV/TV

CC line	μ male	μ female	# male	# female	%diff.	p value
IL-1061	0.068533	0.0856	3	3	24.90	0.569014
IL-111	0.173575	0.1206	4	5	-30.52	**0.011637**
IL-1513	0.23585	0.202075	3	5	-14.32	0.544491
IL-188	0.065843	0.063433	7	3	-3.66	0.92547
IL-1912	0.13682	0.1148	5	4	-16.09	**0.016776**
IL-2156	0.08728	0.049633	5	6	-43.13	**0.049551**
IL-2680	0.06018	0.0509	5	2	-15.42	0.495987
IL-2689	0.09195	0.051775	2	4	-43.69	**0.036892**
IL-3480	0.1169	0.04995	3	2	-57.27	0.084106
IL-3912	0.10892	0.117	5	5	7.42	0.612363
IL-4052	0.09988	0.077429	5	7	-22.48	0.641395
IL-519	0.0873	0.19015	2	4	117.81	0.056429
IL-521	0.062533	0.0507	3	3	-18.92	0.153568
IL-557	0.111933	0.0529	5	4	-52.74	0.162312
IL-72	0.096633	0.065233	3	3	-32.49	**0.021452**

Sex effect of 15 CC lines with >2 observations for both sexes; μ is the sample mean; %diff is the percent difference with positive values indicating females>males; p value is calculated via a two-tailed student's t-test. Total observations are n = 120. Significant effect ($p<0.05$) is indicated in bold. Italic is a border-line significant line at the 5% threshold.

Table S2. Covariate effect on trabecular and cortical traits

Trait	Sex %	logP	Age %	logP	Batch %	logP	Month %	logP	Season %	logP	Year %	logP	Interactions %	logP	Experimenter	logP
BV/TV	-	-	-	-	-	-	-	-	-	-	-	-	-	-	-	-
Tb.N	-	-	-	-	-	-	-	-	-	-	-	-	-	-	-	-
Tb.Th	-	-	2.7	1.5	4.6	2.1	-	-	-	-	-	-	34.70	4.31	3.1	1.70
Conn.D	-	-	-	-	-	-	-	-	-	-	-	-	-		-	-
SMI	-	-	-	-	-	-	-	-	-	-	-	-	-	-	-	-
Tb.Sp	-	-	-	-	-	-	2.5	1.3	-	-	-	-	-	-	-	-
vBMD	-	-	-	-	21.8	8.7	14.2	5.7	15.4	6.1	3.1	1.5	53.92	11.99	-	-
Ct.Th	12.7	5.3	-	-	11.6	4.8	3.0	1.5	8.1	3.5	4.2	2.0	41.07	6.24	5.3	2.52

Effects of covariates (i.e., the degree to which each covariate explains the phenotypic difference). Values were determined by regressing the covariates along with the CC lines, and running an ANOVA test. Note the covariates prominent effect on the cortical traits.

Table S3. Average value of each trait across all the analyzed CC lines

	n	BV/TV (ratio)		Tb.N (mm^{-1})		Tb.Th (mm)		Conn.D (mm^{-3})		Tb.Sp (mm)		SMI (#)		vBMD ($mgHA/cm^3$)		Ct.Th (mm)	
		Mean	SE	Mean	SE	Mean	SE	Mean	SE	Mean	SE	Mean	SE	Mean	SE	Mean	SE
AU18018	4	0.143	0.016	3.72	0.23	0.050	0.002	160.92	13.92	0.24	0.02	2.36	0.16	626.88	18.43	0.225	0.011
AU8010	5	0.124	0.016	3.86	0.40	0.040	0.001	155.57	31.95	0.20	0.01	2.32	0.13	551.81	11.77	0.182	0.007
AU8016	2	0.161	0.001	4.01	0.07	0.051	0.002	149.65	0.13	0.21	0.01	2.14	0.01	777.07	6.11	0.284	0.007
AU8024	3	0.112	0.014	2.89	0.22	0.049	0.003	121.28	8.27	0.27	0.00	2.57	0.12	610.39	14.43	0.206	0.007
AU8048	2	0.049	0.005	1.55	0.14	0.039	0.000	58.77	3.63	0.38	0.01	2.76	0.13	555.64	13.87	0.194	0.002
IL-1061	6	0.077	0.012	1.96	0.35	0.052	0.005	60.10	21.59	0.44	0.07	2.52	0.19	646.52	55.11	0.216	0.021
IL-111	9	0.144	0.011	3.75	0.12	0.048	0.002	153.73	7.73	0.22	0.00	2.12	0.09	537.14	24.44	0.208	0.012
IL-1379	1	0.181	NA	4.10	NA	0.055	NA	152.42	NA	0.25	NA	1.78	NA	709.45	NA	0.240	NA
IL-1452	1	0.155	NA	3.39	NA	0.056	NA	116.43	NA	0.29	NA	1.61	NA	572.46	NA	0.223	NA
IL-1488	3	0.087	0.006	2.06	0.12	0.052	0.002	80.71	9.28	0.69	0.04	1.96	0.11	583.02	4.41	0.213	0.008
IL-1513	6	0.213	0.032	4.76	0.67	0.053	0.001	187.33	32.32	0.21	0.03	1.52	0.33	585.59	41.66	0.210	0.010
IL-16296	1	0.098	NA	2.33	NA	0.053	NA	87.59	NA	0.35	NA	2.54	NA	720.27	NA	0.242	NA
IL-188	10	0.065	0.007	1.61	0.18	0.048	0.001	50.54	6.69	0.51	0.04	2.44	0.09	642.93	13.13	0.211	0.004
IL-1912	9	0.127	0.005	3.49	0.17	0.045	0.002	164.60	17.09	0.28	0.03	2.06	0.09	569.22	21.14	0.197	0.013
IL-2156	11	0.067	0.009	2.17	0.28	0.041	0.002	93.48	14.68	0.32	0.04	2.91	0.05	526.81	12.23	0.184	0.004
IL-2438	2	0.186	0.046	4.21	0.72	0.054	0.004	164.51	22.02	0.24	0.04	1.67	0.22	565.33	3.43	0.180	0.011
IL-2573	3	0.125	0.054	2.85	0.86	0.049	0.006	127.56	31.89	0.41	0.06	1.88	0.40	525.48	37.85	0.188	0.031
IL-2680	7	0.058	0.009	1.79	0.25	0.040	0.001	67.89	12.38	0.37	0.04	2.87	0.11	559.53	15.19	0.192	0.007
IL-2689	6	0.065	0.009	2.20	0.26	0.036	0.001	85.02	13.35	0.30	0.02	2.66	0.05	442.61	10.36	0.163	0.007
IL-2693	3	0.058	0.006	1.32	0.11	0.052	0.001	36.89	7.22	0.73	0.03	2.22	0.33	665.31	29.60	0.219	0.006
IL-3480	5	0.090	0.020	2.45	0.49	0.046	0.001	93.17	22.41	0.32	0.03	2.49	0.27	587.85	31.70	0.210	0.004
IL-3912	10	0.113	0.007	3.03	0.16	0.047	0.001	107.88	9.81	0.25	0.01	2.52	0.07	623.04	26.99	0.207	0.004
IL-4052	12	0.087	0.021	2.04	0.39	0.050	0.002	73.93	18.13	0.46	0.05	2.48	0.19	556.53	28.59	0.193	0.006
IL-519	6	0.156	0.031	3.54	0.46	0.050	0.002	105.43	12.38	0.26	0.02	1.72	0.38	478.08	14.10	0.176	0.013
IL-521	6	0.057	0.004	1.68	0.10	0.044	0.001	46.66	5.05	0.34	0.01	2.97	0.07	554.78	5.73	0.215	0.003
IL-557	7	0.078	0.017	2.04	0.40	0.048	0.001	88.92	25.70	0.35	0.04	2.80	0.19	582.19	37.67	0.211	0.011
IL-57	3	0.076	0.011	1.95	0.24	0.051	0.003	70.86	11.09	0.35	0.05	2.83	0.30	601.17	25.72	0.223	0.010
IL-711	4	0.117	0.007	2.55	0.13	0.058	0.002	81.32	6.91	0.38	0.05	2.13	0.26	692.57	45.58	0.240	0.015
IL-72	6	0.081	0.008	2.24	0.18	0.046	0.001	82.82	10.37	0.37	0.03	2.58	0.08	453.98	8.10	0.184	0.004
IL-785	6	0.106	0.014	2.71	0.32	0.049	0.002	142.65	23.72	0.31	0.02	2.18	0.15	715.44	25.57	0.249	0.010
OR15155	1	0.090	NA	2.83	NA	0.043	NA	98.24	NA	0.23	NA	2.89	NA	519.85	NA	0.203	NA
OR15156	2	0.083	0.014	2.18	0.31	0.048	0.001	80.13	14.14	0.38	0.05	2.16	0.02	562.60	42.27	0.220	0.009
OR3032	2	0.062	0.025	1.54	0.56	0.050	0.003	42.69	15.62	0.59	0.13	2.36	0.19	631.49	12.13	0.217	0.012
OR3393	3	0.210	0.016	4.60	0.28	0.061	0.000	160.95	14.86	0.19	0.01	1.86	0.10	NaN	NA	NaN	NA

Trait means for each line, including number of members and standard error (SE).

Table S4. CC lines associated with genotype at JAX00032223 and trait value (see Fig. 3)
In Allele column, A=TT; B=TC; C=CC

Table S4A

BV/TV	Sex	Allele	Line
0.0854	M	A	IL-2680
0.0592	M	A	IL-2680
0.0433	M	A	IL-188
0.0733	M	A	IL-188
0.0565	M	A	IL-188
0.2414	M	A	IL-4052
0.0732	F	A	IL-4052
0.0384	F	A	IL-4052
0.0532	F	A	IL-4052
0.0642	M	A	IL-188
0.0547	M	A	IL-188
0.0713	M	A	IL-188
0.0976	M	A	IL-188
0.081	M	A	IL-519,
0.0755	M	A	IL-785
0.132	M	A	IL-785
0.1491	M	A	IL-785
0.1311	M	A	IL-785
0.068	M	A	IL-785
0.1064	F	A	IL-188
0.0531	F	A	IL-2680
0.0487	F	A	IL-2680
0.0555	M	A	IL-521,
0.0833	M	A	IL-2680
0.0175	M	A	IL-2680
0.052	F	A	IL-188
0.1124	M	A	IL-4052
0.0263	M	A	IL-4052
0.024	M	A	IL-4052
0.0953	M	A	IL-4052
0.2158	F	A	IL-4052
0.0662	F	A	IL-4052
0.0426	F	A	IL-4052
0.0526	F	A	IL-4052
0.0319	F	A	IL-188
0.0873	M	A	AU8024
0.1345	M	A	AU8024
0.1136	M	A	AU8024

0.0898	M	A	OR15155
0.0691	M	A	OR15156
0.0535	M	A	AU8048
0.0437	M	A	AU8048
0.0968	M	A	IL-57,
0.0376	M	A	OR3032
0.0869	M	A	OR3032
0.0579	M	B	IL-2156
0.1402	M	B	IL-1912
0.1251	F	B	IL-1912,
0.1067	F	B	IL-1912
0.0912	F	B	IL-3912
0.1081	F	B	IL-3912
0.1057	F	B	IL-3912
0.1383	F	B	IL-3912
0.1417	F	B	IL-3912
0.0523	M	B	IL-2156
0.1223	M	B	IL-2156
0.1043	M	B	IL-2156
0.0996	M	B	IL-2156
0.0872	F	B	IL-1061
0.0945	M	B	IL-3912
0.0847	M	B	IL-3912
0.0933	M	B	IL-3912
0.1251	M	B	IL-1912,
0.147	M	B	IL-3912
0.1551	M	B	IL-1452
0.1489	M	B	IL-1912
0.1044	F	B	IL-1912
0.123	F	B	IL-1912
0.1279	M	B	IL-1912
0.1365	M	B	IL-1912
0.1306	M	B	IL-1912,
0.0284	F	B	IL-2156,
0.0572	F	B	IL-2156,
0.0633	F	B	IL-2156,
0.0977	F	B	IL-1061
0.0284	F	B	IL-2156,
0.0572	F	B	IL-2156,
0.0633	F	B	IL-2156,
0.0616	M	B	IL-1061
0.1146	M	B	IL-1061
0.0719	F	B	IL-1061

0.0294	M	B	IL-1061
0.1	M	C	IL-72
0.1054	M	C	IL-72
0.0845	M	C	IL-72
0.0614	F	C	IL-72
0.0628	F	C	IL-72
0.0715	F	C	IL-72
0.1361	F	C	IL-519
0.1259	F	C	IL-519
0.2382	F	C	IL-519
0.0852	M	C	IL-2689
0.0987	M	C	IL-2689
0.0428	F	C	IL-2689
0.0508	F	C	IL-2689
0.0652	F	C	IL-521,
0.0483	F	C	IL-2689
0.1395	M	C	IL-111
0.1329	F	C	IL-111
0.1178	F	C	IL-111
0.1069	F	C	IL-111
0.1328	F	C	IL-111
0.1126	F	C	IL-111
0.2604	F	C	IL-519
0.0936	M	C	IL-519
0.081	M	C	IL-519,
0.0734	F	C	IL-557
0.0315	F	C	IL-557
0.2334	M	C	IL-2573
0.0626	M	C	IL-2573
0.0793	M	C	IL-2573
0.1856	M	C	IL-111
0.1828	M	C	IL-111
0.1864	M	C	IL-111
0.246	F	C	IL-1513
0.1401	M	C	IL-557,
0.0766	M	C	IL-3480
0.1277	M	C	IL-3480
0.1401	M	C	IL-557,
0.0695	M	C	IL-2693
0.0542	M	C	IL-2693
0.049	F	C	IL-2693
0.2491	F	C	IL-1513
0.231	M	C	IL-1513

0.2407	M	C	IL-1513
0.1118	F	C	IL-711
0.1806	M	C	IL-1379
0.2328	F	C	IL-2438
0.1399	F	C	IL-2438
0.1275	M	C	IL-711
0.0556	M	C	IL-557
0.054	F	C	IL-1513
0.0515	F	C	IL-3480
0.0484	F	C	IL-3480
0.1464	M	C	IL-3480
0.0554	F	C	IL-557
0.0513	F	C	IL-557
0.2592	F	C	IL-1513
0.1306	F	C	IL-1912,
0.0993	F	C	IL-711
0.0955	F	C	AU8010
0.0886	F	C	AU8010
0.1324	F	C	AU8010
0.1253	F	C	AU8010
0.1773	M	C	AU8010
0.0975	M	C	IL-16296
0.0742	M	C	IL-57
0.0968	M	C	IL-57,
0.0582	M	C	IL-57
0.0885	M	C	IL-1488
0.0959	M	C	IL-1488
0.0765	M	C	IL-1488
0.2413	M	C	OR3393
0.1969	M	C	OR3393
0.1919	M	C	OR3393
0.1604	M	C	AU8016
0.1622	M	C	AU8016

Table S4B

Tb.N	Sex	Allele	Line
2.5439	M	A	IL-2680
1.7105	M	A	IL-2680
1.0531	M	A	IL-188
1.6507	M	A	IL-188
1.3149	M	A	IL-188
4.6081	M	A	IL-4052

1.9704	F	A	IL-4052
1.1553	F	A	IL-4052
1.4974	F	A	IL-4052
1.9733	M	A	IL-188
1.3665	M	A	IL-188
1.7378	M	A	IL-188
2.6024	M	A	IL-188
2.2233	M	A	IL-785
1.9152	M	A	IL-785
3.0034	M	A	IL-785
3.8995	M	A	IL-785
3.2097	M	A	IL-785
2.0064	M	A	IL-785
2.4034	F	A	IL-188
1.7545	F	A	IL-2680
1.5769	F	A	IL-2680
1.8397	M	A	IL-2680
2.5039	M	A	IL-2680
0.5803	M	A	IL-2680
1.1569	F	A	IL-188
3.2463	M	A	IL-4052
0.7661	M	A	IL-4052
0.5289	M	A	IL-4052
2.404	M	A	IL-4052
4.2434	F	A	IL-4052
1.7172	F	A	IL-4052
1.0348	F	A	IL-4052
1.346	F	A	IL-4052
0.8271	F	A	IL-188
2.5191	M	A	AU8024
3.291	M	A	AU8024
2.8572	M	A	AU8024
2.8289	M	A	OR15155
1.8689	M	A	OR15156
1.6885	M	A	AU8048
1.4021	M	A	AU8048
2.4865	M	A	OR15156
0.9788	M	A	OR3032
2.093	M	A	OR3032
2.0892	M	B	IL-2156
4.0099	M	B	IL-1912
3.4716	F	B	IL-1912
2.9866	F	B	IL-1912

2.4727	F	B	IL-3912
2.9892	F	B	IL-3912
2.7947	F	B	IL-3912
3.2645	F	B	IL-3912
3.4381	F	B	IL-3912
1.4351	M	B	IL-2156
3.6839	M	B	IL-2156
2.9917	M	B	IL-2156
3.0093	M	B	IL-2156
2.2399	F	B	IL-1061
2.6759	M	B	IL-3912
2.5375	M	B	IL-3912
2.659	M	B	IL-3912
3.4587	M	B	IL-3912
4.0203	M	B	IL-3912
3.3859	M	B	IL-1452
4.4259	M	B	IL-1912
3.214	F	B	IL-1912
3.4354	F	B	IL-1912
2.9774	M	B	IL-1912
3.0316	M	B	IL-1912
3.8883	M	B	IL-1912
0.7012	F	B	IL-2156,
2.2381	F	B	IL-2156,
2.3833	F	B	IL-2156,
1.894	F	B	IL-1061
0.7012	F	B	IL-2156,
2.2381	F	B	IL-2156,
2.3833	F	B	IL-2156,
2.0319	M	B	IL-1061
3.3668	M	B	IL-1061
1.303	F	B	IL-1061
0.901	M	B	IL-1061
2.8162	M	C	IL-72
2.641	M	C	IL-72
2.255	M	C	IL-72
1.8208	F	C	IL-72
1.7379	F	C	IL-72
2.1622	F	C	IL-72
3.2392	F	C	IL-519
3.2207	F	C	IL-519
4.9549	F	C	IL-519
2.6746	M	C	IL-2689

3.2278	M	C	IL-2689
1.5908	F	C	IL-2689
1.8642	F	C	IL-2689
2.1089	F	C	IL-2689
1.7532	F	C	IL-2689
3.7129	M	C	IL-111
3.911	F	C	IL-111
3.4928	F	C	IL-111
3.2452	F	C	IL-111
3.6499	F	C	IL-111
3.3449	F	C	IL-111
4.8652	F	C	IL-519
2.7026	M	C	IL-519
2.2594	M	C	IL-519
1.9692	F	C	IL-557
0.8359	F	C	IL-557
4.5509	M	C	IL-2573
1.7484	M	C	IL-2573
2.2464	M	C	IL-2573
4.2742	M	C	IL-111
4.1007	M	C	IL-111
4.0385	M	C	IL-111
5.6775	F	C	IL-1513
3.5312	M	C	IL-557,
2.186	M	C	IL-3480
3.4176	M	C	IL-3480
3.5312	M	C	IL-557,
1.5113	M	C	IL-2693
1.3173	M	C	IL-2693
1.1432	F	C	IL-2693
5.2888	F	C	IL-1513
4.9802	M	C	IL-1513
5.5807	M	C	IL-1513
2.6447	F	C	IL-711
4.0964	M	C	IL-1379
4.9249	F	C	IL-2438
3.4874	F	C	IL-2438
2.7551	M	C	IL-711
1.5931	M	C	IL-557
1.4562	F	C	IL-1513
1.5984	F	C	IL-3480
1.3042	F	C	IL-3480
3.7662	M	C	IL-3480

1.4111	F	C	IL-557
1.4307	F	C	IL-557
5.6059	F	C	IL-1513
2.6216	F	C	IL-711
2.1644	F	C	IL-711
3.1154	F	C	AU8010
3.0734	F	C	AU8010
4.0688	F	C	AU8010
3.767	F	C	AU8010
5.2628	M	C	AU8010
2.3262	M	C	IL-16296
1.719	M	C	IL-57
2.435	M	C	IL-57
1.6892	M	C	IL-57
1.9772	M	C	IL-1488
2.2995	M	C	IL-1488
1.9175	M	C	IL-1488
5.1701	M	C	OR3393
4.3083	M	C	OR3393
4.324	M	C	OR3393
4.0766	M	C	AU8016
3.9361	M	C	AU8016

Table S4C

Tb.Th	Sex	Allele	Line	
0.0431	M	A	IL-2573,	IL-2680
0.0435	M	A	IL-2680	
0.0465	M	A	IL-188	
0.05	M	A	IL-188,	IL-4052
0.0486	M	A	IL-188,	IL-4052
0.066	M	A	IL-4052	
0.0486	F	A	IL-188,	IL-4052
0.0408	F	A	IL-4052,	IL-4052
0.0466	F	A	IL-4052	
0.0414	M	A	IL-188	
0.0484	M	A	IL-188	
0.0485	M	A	IL-3912,	IL-188,
0.0488	M	A	IL-72,	IL-188
0.0459	M	A	IL-72,	IL-785,
0.051	M	A	IL-785	
0.055	M	A	IL-785	
0.0493	M	A	IL-785	

0.0508	M	A	IL-785,	IL-1513
0.0419	M	A	IL-785	
0.0533	F	A	IL-3912,	IL-188,
0.0377	F	A	IL-2680	
0.0405	F	A	IL-2680	
0.0393	M	A	AU8048,	IL-2680
0.0411	M	A	IL-2680	
0.0369	M	A	IL-2680	
0.0529	F	A	IL-188	
0.0449	M	A	IL-4052	
0.0408	M	A	IL-4052,	IL-4052
0.0551	M	A	IL-1379,	IL-4052
0.0492	M	A	IL-4052	
0.0599	F	A	IL-4052	
0.05	F	A	IL-188,	IL-4052
0.0522	F	A	IL-57,	OR3032,
0.0507	F	A	IL-2693,	IL-2438,
0.0461	F	A	IL-3912,	IL-3912,
0.0437	M	A	AU8010,	AU8024
0.0525	M	A	IL-2693,	AU8024
0.052	M	A	IL-1488,	AU8024
0.0429	M	A	IL-1912,	IL-2156,
0.047	M	A	OR15156	
0.0393	M	A	AU8048,	IL-2680
0.0394	M	A	AU8048	
0.0498	M	A	OR15156	
0.0471	M	A	OR3032	
0.0522	M	A	IL-57,	OR3032,
0.0339	M	B	IL-2156	
0.0429	M	B	IL-1912,	IL-2156,
0.0426	F	B	IL-1912	
0.0425	F	B	IL-1912,	IL-2573
0.0485	F	B	IL-3912,	IL-188,
0.0457	F	B	IL-3912	
0.0472	F	B	IL-3912,	IL-557
0.0547	F	B	IL-3912	
0.0533	F	B	IL-3912,	IL-188,
0.0497	M	B	IL-2156,	AU8016
0.0429	M	B	IL-1912,	IL-2156,
0.0458	M	B	IL-2156,	IL-3912
0.0428	M	B	IL-2156	
0.0489	F	B	IL-1061	
0.0458	M	B	IL-2156,	IL-3912

0.0418	M	B	IL-3912	
0.0454	M	B	IL-521,	IL-3912
0.0461	M	B	IL-3912,	IL-3912,
0.0461	M	B	IL-3912,	IL-3912,
0.0557	M	B	IL-1452	
0.041	M	B	IL-1912	
0.04	F	B	IL-1912	
0.0438	F	B	IL-72,	IL-1912,
0.0539	M	B	IL-519,	IL-1912
0.0543	M	B	IL-1912	
0.0423	M	B	IL-1912	
0.0495	F	B	IL-2156,	IL-2156
0.0319	F	B	IL-2156,	IL-2156
0.0344	F	B	IL-2156,	IL-2156
0.0688	F	B	IL-1061	
0.0495	F	B	IL-2156,	IL-2156
0.0319	F	B	IL-2156,	IL-2156
0.0344	F	B	IL-2156,	IL-2156
0.0403	M	B	IL-1061	
0.045	M	B	IL-1061	
0.0655	F	B	IL-1061	
0.0413	M	B	AU8010,	IL-1061
0.0444	M	C	IL-72	
0.0499	M	C	IL-72	
0.0488	M	C	IL-72,	IL-188
0.0438	F	C	IL-72,	IL-1912,
0.0459	F	C	IL-72,	IL-785,
0.0427	F	C	IL-72	
0.0502	F	C	IL-519,	AU18018
0.0463	F	C	IL-519,	IL-111,
0.0539	F	C	IL-519,	IL-1912
0.0404	M	C	IL-2689	
0.0373	M	C	IL-2689	
0.0323	F	C	IL-2689	
0.0327	F	C	IL-2689	
0.0397	F	C	IL-2689	
0.0333	F	C	IL-2689	
0.0463	M	C	IL-519,	IL-111,
0.0433	F	C	IL-111,	IL-521
0.044	F	C	IL-111,	IL-3480,
0.0443	F	C	IL-111	
0.0462	F	C	IL-111	
0.0439	F	C	IL-111,	IL-519

0.06	F	C	IL-519	
0.0439	M	C	IL-111,	IL-519
0.0455	M	C	IL-519	
0.0463	F	C	IL-519,	IL-111,
0.0472	F	C	IL-3912,	IL-557
0.0607	M	C	IL-2573	
0.0425	M	C	IL-1912,	IL-2573
0.0431	M	C	IL-2573,	IL-2680
0.0545	M	C	IL-111	
0.0558	M	C	IL-111	
0.0574	M	C	IL-111	
0.0504	F	C	IL-1513,	IL-557,
0.0504	M	C	IL-1513,	IL-557,
0.044	M	C	IL-111,	IL-3480,
0.046	M	C	IL-3480	
0.0504	M	C	IL-1513,	IL-557,
0.0525	M	C	IL-2693,	AU8024
0.054	M	C	IL-2693	
0.0507	F	C	IL-2693,	IL-2438,
0.0572	F	C	IL-1513	
0.0564	M	C	IL-1513	
0.0516	M	C	IL-1513	
0.0533	F	C	IL-3912,	IL-188,
0.0551	M	C	IL-1379,	IL-4052
0.0578	F	C	IL-2438	
0.0507	F	C	IL-2693,	IL-2438,
0.0563	M	C	IL-711	
0.0483	M	C	IL-557	
0.0508	F	C	IL-785,	IL-1513
0.0416	F	C	IL-3480	
0.0477	F	C	IL-3480	
0.0485	M	C	IL-3912,	IL-188,
0.0509	F	C	IL-557	
0.0453	F	C	IL-557	
0.053	F	C	IL-1513	
0.0649	F	C	IL-711	
0.0591	F	C	IL-711	
0.0392	F	C	AU8010	
0.0358	F	C	AU8010	
0.0413	F	C	AU8010,	IL-1061
0.0421	F	C	AU8010	
0.0437	M	C	AU8010,	AU8024
0.0534	M	C	IL-16296	

0.0549	M	C	IL-57	
0.0522	M	C	IL-57,	OR3032,
0.0459	M	C	IL-72,	IL-785,
0.0556	M	C	IL-1488	
0.052	M	C	IL-1488,	AU8024
0.0496	M	C	IL-1488	
0.0621	M	C	OR3393	
0.0612	M	C	OR3393	
0.0605	M	C	OR3393	
0.0497	M	C	IL-2156,	AU8016
0.0528	M	C	AU8016	

Table S4D

Conn.D	Sex	Allele	Line
105.1802	M	A	IL-2680
71.1679	M	A	IL-2680
31.4851	M	A	IL-188
45.3736	M	A	IL-188
43.7289	M	A	IL-188
197.3656	M	A	IL-4052
67.2506	F	A	IL-4052
33.246	F	A	IL-4052
46.9579	F	A	IL-4052
62.5413	M	A	IL-188
52.4396	M	A	IL-188
62.0701	M	A	IL-188
76.3582	M	A	IL-188
108.9201	M	A	IL-785
79.6112	M	A	IL-785
153.6653	M	A	IL-785
234.8736	M	A	IL-785
178.8657	M	A	IL-785
99.9562	M	A	IL-785
83.3347	F	A	IL-188
43.8273	F	A	IL-2680
46.9229	F	A	IL-2680
78.9733	M	A	IL-2680
108.5448	M	A	IL-2680
20.5793	M	A	IL-2680
33.2201	F	A	IL-188
143.8799	M	A	IL-4052
16.6288	M	A	IL-4052

10.9829	M	A	IL-4052	
86.9122	M	A	IL-4052	
168.1929	F	A	IL-4052	
61.1745	F	A	IL-4052	
24.3196	F	A	IL-4052	
30.205	F	A	IL-4052	
14.8824	F	A	IL-188	
106.5821	M	A	AU8024	
135.1928	M	A	AU8024	
122.0755	M	A	AU8024	
98.2431	M	A	OR15155	
65.9885	M	A	OR15156	
62.3924	M	A	AU8048	
55.1387	M	A	AU8048	
94.2625	M	A	OR15156	
27.0639	M	A	OR3032	
58.3123	M	A	OR3032	
80.5979	M	B	IL-2156	
206.4792	M	B	IL-1912	
167.216	F	B	IL-1912	
143.2627	F	B	IL-1912	
70.3262	F	B	IL-3912	
104.4889	F	B	IL-3912	
86.2983	F	B	IL-3912	
106.089	F	B	IL-3912	
124.602	F	B	IL-3912	
29.9469	M	B	IL-2156	
166.7973	M	B	IL-2156	
109.9193	M	B	IL-2156	
130.9429	M	B	IL-2156	
79.1354	F	B	IL-1061	
94.0339	M	B	IL-3912	
89.1647	M	B	IL-3912	
88.7592	M	B	IL-3912	
139.3613	M	B	IL-3912	
175.6634	M	B	IL-3912	
116.4258	M	B	IL-1452	
259.6617	M	B	IL-1912	
145.6863	F	B	IL-1912	
151.7723	F	B	IL-1912	
108.3193	M	B	IL-1912	
96.4091	M	B	IL-1912	
202.5703	M	B	IL-1912	IL-2156

21.6866	F	B	IL-2156,	IL-2156
118.2116	F	B	IL-2156,	IL-2156
115.1224	F	B	IL-2156,	
31.1473	F	B	IL-1061	IL-2156
21.6866	F	B	IL-2156,	IL-2156
118.2116	F	B	IL-2156,	IL-2156
115.1224	F	B	IL-2156,	
66.2345	M	B	IL-1061	
153.5026	M	B	IL-1061	
17.7388	F	B	IL-1061	
12.8537	M	B	IL-1061	
119.6713	M	C	IL-72	
99.5861	M	C	IL-72	
84.8398	M	C	IL-72	
49.5742	F	C	IL-72	
61.02	F	C	IL-72	
82.1996	F	C	IL-72	
101.0982	F	C	IL-519	
113.0052	F	C	IL-519	
145.8939	F	C	IL-519	
96.1453	M	C	IL-2689	
146.0754	M	C	IL-2689	
65.0352	F	C	IL-2689	
64.9911	F	C	IL-2689	
78.2814	F	C	IL-2689	
59.6182	F	C	IL-2689	
145.6938	M	C	IL-111	
207.8187	F	C	IL-111	
158.7865	F	C	IL-111	
143.8708	F	C	IL-111	
158.0172	F	C	IL-111	
132.8133	F	C	IL-111	
125.7239	F	C	IL-519	
87.9026	M	C	IL-519	
58.9263	M	C	IL-519	
94.3199	F	C	IL-557	
27.5189	F	C	IL-557	
187.0529	M	C	IL-2573	
77.89	M	C	IL-2573	
117.7311	M	C	IL-2573	
162.8908	M	C	IL-111	
143.2605	M	C	IL-111	
130.3896	M	C	IL-111	

253.6898	F	C	IL-1513	IL-557
183.344	M	C	IL-557,	
109.3634	M	C	IL-3480	
134.0649	M	C	IL-3480	IL-557
183.344	M	C	IL-557,	
51.2884	M	C	IL-2693	
28.6856	M	C	IL-2693	
30.7083	F	C	IL-2693	
199.2888	F	C	IL-1513	
194.8097	M	C	IL-1513	
236.0602	M	C	IL-1513	
79.9398	F	C	IL-711	
152.4168	M	C	IL-1379	
186.5265	F	C	IL-2438	
142.4856	F	C	IL-2438	
93.9514	M	C	IL-711	
38.1719	M	C	IL-557	
32.5951	F	C	IL-1513	
44.6507	F	C	IL-3480	
35.4067	F	C	IL-3480	
142.3585	M	C	IL-3480	
56.8558	F	C	IL-557	
38.8677	F	C	IL-557	
207.5341	F	C	IL-1513	
88.878	F	C	IL-711	
62.5068	F	C	IL-711	
101.7011	F	C	AU8010	
92.2759	F	C	AU8010	
177.5241	F	C	AU8010	
138.0187	F	C	AU8010	
268.3448	M	C	AU8010	
87.5885	M	C	IL-16296	
80.0751	M	C	IL-57	
83.7252	M	C	IL-57	
48.7778	M	C	IL-57	
68.211	M	C	IL-1488	
98.8458	M	C	IL-1488	
75.0742	M	C	IL-1488	
190.0075	M	C	OR3393	
140.9733	M	C	OR3393	
151.8749	M	C	OR3393	
149.5133	M	C	AU8016	
149.7832	M	C	AU8016	

Table S4E

SMI	Sex	Allele	Line
2.7646	M	A	IL-2680
2.5287	M	A	IL-2680
2.3211	M	A	IL-188
2.3623	M	A	IL-188
2.3228	M	A	IL-188
1.4329	M	A	IL-4052
2.6172	F	A	IL-4052
3.2404	F	A	IL-4052
2.7093	F	A	IL-4052
3.0319	M	A	IL-188
2.4351	M	A	IL-188
2.1512	M	A	IL-188
2.7117	M	A	IL-188
2.4559	M	A	IL-785
2.4071	M	A	IL-785
1.7465	M	A	IL-785
2.0682	M	A	IL-785
1.8238	M	A	IL-785
2.6081	M	A	IL-785
2.0109	F	A	IL-188
3.111	F	A	IL-2680
3.0693	F	A	IL-2680
2.9191	M	A	IL-2680
2.51	M	A	IL-2680
3.2076	M	A	IL-2680
2.3871	F	A	IL-188
2.7982	M	A	IL-4052
3.0801	M	A	IL-4052
3.2188	M	A	IL-4052
2.0739	M	A	IL-4052
1.1824	F	A	IL-4052
2.4006	F	A	IL-4052
2.4185	F	A	IL-4052
2.6431	F	A	IL-4052
2.6897	F	A	IL-188
2.8107	M	A	AU8024
2.4186	M	A	AU8024
2.4861	M	A	AU8024
2.8918	M	A	OR15155

2.1842	M	A	OR15156
2.6324	M	A	AU8048
2.8947	M	A	AU8048
2.1369	M	A	OR15156
2.5543	M	A	OR3032
2.1748	M	A	OR3032
2.8969	M	B	IL-2156
2.155	M	B	IL-1912
1.9702	F	B	IL-1912
2.2993	F	B	IL-1912
2.7689	F	B	IL-3912
2.4995	F	B	IL-3912
2.4564	F	B	IL-3912
2.2953	F	B	IL-3912
2.2504	F	B	IL-3912
3.1993	M	B	IL-2156
2.5753	M	B	IL-2156
2.9502	M	B	IL-2156
2.8986	M	B	IL-2156
2.2094	F	B	IL-1061
2.7875	M	B	IL-3912
2.8075	M	B	IL-3912
2.7395	M	B	IL-3912
2.3907	M	B	IL-3912
2.2427	M	B	IL-3912
1.6082	M	B	IL-1452
2.0793	M	B	IL-1912
2.2625	F	B	IL-1912
2.2036	F	B	IL-1912
1.7748	M	B	IL-1912
1.4891	M	B	IL-1912
2.2874	M	B	IL-1912
3.0517	F	B	IL-2156,
2.7929	F	B	IL-2156,
2.9048	F	B	IL-2156,
2.3364	F	B	IL-1061
3.0517	F	B	IL-2156,
2.7929	F	B	IL-2156,
2.9048	F	B	IL-2156,
3.1399	M	B	IL-1061
2.5612	M	B	IL-1061
1.8904	F	B	IL-1061
2.9791	M	B	IL-1061

2.4384	M	C	IL-72
2.3172	M	C	IL-72
2.4897	M	C	IL-72
2.8724	F	C	IL-72
2.6942	F	C	IL-72
2.6452	F	C	IL-72
1.7472	F	C	IL-519
1.722	F	C	IL-519
0.7013	F	C	IL-519
2.7401	M	C	IL-2689
2.4203	M	C	IL-2689
2.621	F	C	IL-2689
2.7251	F	C	IL-2689
2.6957	F	C	IL-2689
2.7746	F	C	IL-2689
2.2135	M	C	IL-111
2.0221	F	C	IL-111
2.4407	F	C	IL-111
2.4671	F	C	IL-111
2.1866	F	C	IL-111
2.3524	F	C	IL-111
0.6331	F	C	IL-519
2.6065	M	C	IL-519
2.9018	M	C	IL-519
2.7988	F	C	IL-557
3.1347	F	C	IL-557
1.0678	M	C	IL-2573
2.3149	M	C	IL-2573
2.2462	M	C	IL-2573
1.8466	M	C	IL-111
1.791	M	C	IL-111
1.7734	M	C	IL-111
1.2445	F	C	IL-1513
2.0884	M	C	IL-557,
2.36	M	C	IL-3480
2.0325	M	C	IL-3480
2.0884	M	C	IL-557,
1.5853	M	C	IL-2693
2.6842	M	C	IL-2693
2.403	F	C	IL-2693
1.2643	F	C	IL-1513
1.3149	M	C	IL-1513
1.2246	M	C	IL-1513

2.5894	F	C	IL-711
1.7815	M	C	IL-1379
1.4474	F	C	IL-2438
1.8958	F	C	IL-2438
1.4197	M	C	IL-711
3.0773	M	C	IL-557
3.1504	F	C	IL-1513
2.9538	F	C	IL-3480
3.2388	F	C	IL-3480
1.8524	M	C	IL-3480
2.9966	F	C	IL-557
3.3919	F	C	IL-557
0.9375	F	C	IL-1513
2.0412	F	C	IL-711
2.4542	F	C	IL-711
2.564	F	C	AU8010
2.6497	F	C	AU8010
2.1611	F	C	AU8010
2.2734	F	C	AU8010
1.964	M	C	AU8010
2.5402	M	C	IL-16296
2.345	M	C	IL-57
2.7752	M	C	IL-57
3.3788	M	C	IL-57
1.9088	M	C	IL-1488
1.7888	M	C	IL-1488
2.1759	M	C	IL-1488
1.654	M	C	OR3393
1.9548	M	C	OR3393
1.9606	M	C	OR3393
2.1548	M	C	AU8016
2.1261	M	C	AU8016

Table S4F

Sp	Sex	Allele	Line
0.266	M	A	IL-2680
0.4887	M	A	IL-2680
0.7139	M	A	IL-188
0.5608	M	A	IL-188
0.5049	M	A	IL-188
0.2431	M	A	IL-4052
0.3837	F	A	IL-4052

0.4598	F	A	IL-4052
0.4386	F	A	IL-4052
0.2992	M	A	IL-188
0.53	M	A	IL-188
0.5813	M	A	IL-188
0.2797	M	A	IL-188
0.3658	M	A	IL-785
0.2588	M	A	IL-785
0.3648	M	A	IL-785
0.2435	M	A	IL-785
0.3062	M	A	IL-785
0.3386	M	A	IL-785
0.5004	F	A	IL-188
0.3064	F	A	IL-2680
0.3666	F	A	IL-2680
0.3342	M	A	IL-2680
0.3012	M	A	IL-2680
0.5191	M	A	IL-2680
0.545	F	A	IL-188
0.2171	M	A	IL-4052
0.4524	M	A	IL-4052
0.6385	M	A	IL-4052
0.4645	M	A	IL-4052
0.3118	F	A	IL-4052
0.5689	F	A	IL-4052
0.7997	F	A	IL-4052
0.4917	F	A	IL-4052
0.611	F	A	IL-188
0.2709	M	A	AU8024
0.2616	M	A	AU8024
0.2758	M	A	AU8024
0.2314	M	A	OR15155
0.422	M	A	OR15156
0.3704	M	A	AU8048
0.3885	M	A	AU8048
0.3297	M	A	OR15156
0.7157	M	A	OR3032
0.4555	M	A	OR3032
0.2855	M	B	IL-2156
0.2117	M	B	IL-1912,
0.2653	F	B	IL-1912
0.2869	F	B	IL-1912
0.2824	F	B	IL-3912

0.2574	F	B	IL-3912
0.2686	F	B	IL-3912
0.259	F	B	IL-3912
0.2545	F	B	IL-3912
0.3733	M	B	IL-2156
0.1955	M	B	IL-2156
0.2194	M	B	IL-2156
0.2241	M	B	IL-2156,
0.4633	F	B	IL-1061
0.2518	M	B	IL-3912
0.2595	M	B	IL-3912
0.2507	M	B	IL-3912
0.2326	M	B	IL-3912
0.2045	M	B	IL-3912
0.2921	M	B	IL-1452
0.1913	M	B	IL-1912
0.2557	F	B	IL-1912
0.2548	F	B	IL-1912
0.3998	M	B	IL-1912
0.4395	M	B	IL-1912
0.2106	M	B	IL-1912
0.5957	F	B	IL-2156,
0.2795	F	B	IL-2156,
0.2633	F	B	IL-2156,
0.4166	F	B	IL-1061
0.5957	F	B	IL-2156,
0.2795	F	B	IL-2156,
0.2633	F	B	IL-2156,
0.2889	M	B	IL-1061
0.2339	M	B	IL-1061
0.5782	F	B	IL-1061
0.6517	M	B	IL-1061
0.2937	M	C	IL-72
0.3249	M	C	IL-72
0.4534	M	C	IL-72
0.3364	F	C	IL-72
0.4939	F	C	IL-72
0.3405	F	C	IL-72
0.3058	F	C	IL-519
0.3027	F	C	IL-519
0.2198	F	C	IL-519
0.2519	M	C	IL-2689
0.2228	M	C	IL-2689

0.3868	F	C	IL-2689
0.3123	F	C	IL-2689
0.3032	F	C	IL-2689
0.3126	F	C	IL-2689
0.2181	M	C	IL-111
0.2182	F	C	IL-111
0.221	F	C	IL-111
0.2261	F	C	IL-111
0.2211	F	C	IL-111
0.2325	F	C	IL-111
0.2111	F	C	IL-519
0.2638	M	C	IL-519
0.2863	M	C	IL-519
0.2948	F	C	IL-557
0.5688	F	C	IL-557
0.286	M	C	IL-2573
0.504	M	C	IL-2573
0.4375	M	C	IL-2573,
0.2124	M	C	IL-111
0.2156	M	C	IL-111
0.2264	M	C	IL-111
0.1682	F	C	IL-1513
0.2426	M	C	IL-557,
0.4375	M	C	IL-2573,
0.2753	M	C	IL-3480
0.2426	M	C	IL-557,
0.7635	M	C	IL-2693
0.7014	M	C	IL-2693
0.1819	F	C	IL-1513
0.198	M	C	IL-1513
0.169	M	C	IL-1513
0.3068	F	C	IL-711
0.2514	M	C	IL-1379
0.1985	F	C	IL-2438
0.2845	F	C	IL-2438
0.5078	M	C	IL-711
0.3616	M	C	IL-557
0.3852	F	C	IL-1513
0.3582	F	C	IL-3480
0.3102	F	C	IL-3480
0.2355	M	C	IL-3480
0.4018	F	C	IL-557
0.3344	F	C	IL-557

0.1697	F	C	IL-1513
0.3286	F	C	IL-711
0.3785	F	C	IL-711
0.2165	F	C	AU8010
0.2094	F	C	AU8010
0.1999	F	C	AU8010
0.2051	F	C	AU8010
0.1603	M	C	AU8010
0.3485	M	C	IL-16296
0.4399	M	C	IL-57
0.2903	M	C	IL-57
0.3107	M	C	IL-57
0.7023	M	C	IL-1488
0.6222	M	C	IL-1488
0.7419	M	C	IL-1488
0.173	M	C	OR3393
0.1993	M	C	OR3393
0.1969	M	C	OR3393
0.2013	M	C	AU8016
0.2167	M	C	AU8016

Table S4G

Ct.Th	Sex	Allele	Line
0.2201	M	A	IL-2680
0.1868	M	A	IL-2680
0.217	M	A	IL-188
0.2232	M	A	IL-188
0.2103	M	A	IL-188
0.213	M	A	IL-4052
0.1792	F	A	IL-1912,
0.1815	F	A	IL-4052
0.1984	F	A	IL-188,
0.1951	M	A	IL-188
0.214	M	A	IL-188
0.1965	M	A	IL-188
0.1984	M	A	IL-188,
0.2339	M	A	IL-785
0.2722	M	A	IL-785
0.2474	M	A	IL-785
0.2729	M	A	IL-785
0.2196	M	A	IL-785
0.2279	F	A	IL-188

0.1872	F	A	IL-2680
0.1821	F	A	IL-2680
0.1855	M	A	IL-2680
0.2106	M	A	IL-2680
0.1688	M	A	IL-2680
0.2261	F	A	IL-188
0.1991	M	A	IL-521,
0.2066	M	A	IL-1912,
0.1746	M	A	IL-4052
0.2258	F	A	IL-4052
0.1612	F	A	IL-4052
0.1776	F	A	IL-4052
0.2063	F	A	IL-4052
0.198	F	A	IL-188
0.1961	M	A	AU8024
0.22	M	A	AU8024
0.2017	M	A	AU8024
0.2029	M	A	OR15155
0.2111	M	A	OR15156
0.1967	M	A	AU8048
0.1922	M	A	AU8048
0.2283	M	A	IL-2693,
0.2054	M	A	OR3032
0.2291	M	A	OR3032
0.1778	M	B	IL-2156
0.1857	M	B	IL-1912
0.1607	F	B	IL-1912
0.1663	F	B	IL-1912
0.1982	F	B	IL-3912
0.2019	F	B	IL-3912
0.1964	F	B	IL-3912
0.2056	F	B	IL-3912
0.2008	F	B	IL-3912
0.1962	M	B	IL-2156
0.1993	M	B	IL-2156
0.2064	M	B	IL-2156
0.1917	M	B	IL-2156
0.2209	M	B	IL-3912
0.2022	M	B	IL-3912
0.2302	M	B	IL-3912
0.2228	M	B	IL-1452
0.2066	M	B	IL-1912,
0.1752	F	B	IL-1912

0.1792	F	B	IL-1912,
0.2602	M	B	IL-1912
0.2642	M	B	IL-1912
0.1714	M	B	IL-1912
0.1853	F	B	IL-2156,
0.161	F	B	IL-2156,
0.1823	F	B	IL-2156,
0.2525	F	B	IL-1061
0.1853	F	B	IL-2156,
0.161	F	B	IL-2156,
0.1823	F	B	IL-2156,
0.1977	M	B	IL-1061
0.1987	M	B	IL-1061
0.2757	F	B	IL-1061
0.1565	M	B	IL-1061
0.1994	M	C	IL-72
0.1795	M	C	IL-72
0.1902	M	C	IL-72
0.1802	F	C	IL-72
0.1756	F	C	IL-72
0.1765	F	C	IL-72
0.1471	F	C	IL-519
0.1766	F	C	IL-519
0.184	M	C	IL-2689
0.1829	M	C	IL-2689
0.1495	F	C	IL-2689
0.1443	F	C	IL-2689
0.1676	F	C	IL-2689
0.147	F	C	IL-2689
0.2328	M	C	IL-111
0.1761	F	C	IL-111
0.189	F	C	IL-111
0.1728	F	C	IL-111
0.174	F	C	IL-111
0.1875	F	C	IL-111
0.1711	F	C	IL-519
0.2087	M	C	IL-519
0.193	F	C	IL-557
0.1783	F	C	IL-557
0.2198	M	C	IL-2573
0.1571	M	C	IL-2573
0.2666	M	C	IL-111
0.2365	M	C	IL-111

0.2379	M	C	IL-111
0.1739	F	C	IL-1513
0.2047	M	C	IL-3480
0.2057	M	C	IL-3480
0.2202	M	C	IL-2693
0.2092	M	C	IL-2693
0.2283	F	C	IL-2693,
0.2411	F	C	IL-1513
0.1912	M	C	IL-1513
0.2191	M	C	IL-1513
0.1972	F	C	IL-711
0.2399	M	C	IL-1379
0.1919	F	C	IL-2438
0.169	F	C	IL-2438
0.2637	M	C	IL-711
0.2186	M	C	IL-557
0.2213	F	C	IL-1513
0.2238	F	C	IL-3480
0.2014	F	C	IL-3480
0.2151	M	C	IL-3480
0.2308	F	C	IL-557
0.2321	F	C	IL-557
0.2148	F	C	IL-1513
0.2507	F	C	IL-711
0.2486	F	C	IL-711
0.1816	F	C	AU8010
0.1619	F	C	AU8010
0.1843	F	C	AU8010
0.18	F	C	AU8010
0.2045	M	C	AU8010
0.2417	M	C	IL-16296
0.2058	M	C	IL-57
0.2221	M	C	IL-57
0.2413	M	C	IL-57
0.2282	M	C	IL-1488
0.2077	M	C	IL-1488
0.2026	M	C	IL-1488
0.291	M	C	AU8016
0.2761	M	C	AU8016

Table S4H

vBMD	Sex	Allele	Line

526.2495	M	A	IL-2680
543.1779	M	A	IL-2680
591.0477	M	A	IL-188
596.0321	M	A	IL-188
611.6439	M	A	IL-188
468.4108	M	A	IL-4052
452.1408	F	A	IL-4052
490.4178	F	A	IL-4052
641.9269	M	A	IL-188
641.1745	M	A	IL-188
622.4592	M	A	IL-188
695.6275	M	A	IL-188
662.3011	M	A	IL-785
692.7051	M	A	IL-785
773.7803	M	A	IL-785
779.7993	M	A	IL-785
668.6361	M	A	IL-785
684.8121	F	A	IL-188
571.2038	F	A	IL-2680
555.2159	F	A	IL-2680
630.4532	M	A	IL-2680
582.1132	M	A	IL-2680
508.2866	M	A	IL-2680
710.769	F	A	IL-188
603.5558	M	A	IL-4052
656.0339	M	A	IL-4052
535.184	M	A	IL-4052
701.1763	F	A	IL-4052
584.6525	F	A	IL-4052
517.2211	F	A	IL-4052
633.8031	F	A	IL-188
622.5533	M	A	AU8024
626.9734	M	A	AU8024
581.643	M	A	AU8024
519.8544	M	A	OR15155
520.3246	M	A	OR15156
569.511	M	A	AU8048
541.7672	M	A	AU8048
604.8725	M	A	OR15156
619.3557	M	A	OR3032
643.6198	M	A	OR3032
506.1235	M	B	IL-2156
541.1089	M	B	IL-1912

513.4592	F	B	IL-1912
524.4626	F	B	IL-1912
685.4705	F	B	IL-3912
707.9476	F	B	IL-3912
651.3315	F	B	IL-3912
675.6896	F	B	IL-3912
615.6879	F	B	IL-3912
504.6204	M	B	IL-2156
493.9379	M	B	IL-2156
505.99	M	B	IL-2156
477.7773	M	B	IL-2156
500.6944	M	B	IL-3912
517.9507	M	B	IL-3912
629.5233	M	B	IL-3912
572.4587	M	B	IL-1452
628.1961	M	B	IL-1912
524.1805	F	B	IL-1912
550.8898	F	B	IL-1912,
644.8423	M	B	IL-1912
678.6991	M	B	IL-1912
517.127	M	B	IL-1912
599.4178	F	B	IL-2156,
510.4497	F	B	IL-2156,
543.366	F	B	IL-2156,
716.9761	F	B	IL-1061
599.4178	F	B	IL-2156,
510.4497	F	B	IL-2156,
543.366	F	B	IL-2156,
626.7854	M	B	IL-1061
595.1857	M	B	IL-1061
809.2358	F	B	IL-1061
484.3961	M	B	IL-1061
461.8276	M	C	IL-72
455.9026	M	C	IL-72
434.8362	F	C	IL-72
479.1322	F	C	IL-72
438.2219	F	C	IL-72
436.0588	F	C	IL-519
488.7249	F	C	IL-519
474.1477	M	C	IL-2689
459.4764	M	C	IL-2689
428.5351	F	C	IL-2689
402.5782	F	C	IL-2689

437.6576	F	C	IL-2689
453.2693	F	C	IL-2689
542.9898	M	C	IL-111
475.4644	F	C	IL-111
506.8759	F	C	IL-111
469.4454	F	C	IL-111
493.8975	F	C	IL-111
496.3427	F	C	IL-519
491.1989	M	C	IL-519
502.977	F	C	IL-557
478.2338	F	C	IL-557
563.3284	M	C	IL-2573
487.638	M	C	IL-2573
664.2159	M	C	IL-111
614.7474	M	C	IL-111
529.4549	M	C	IL-111
443.2646	F	C	IL-1513
533.107	M	C	IL-3480
559.6361	M	C	IL-3480
656.1279	M	C	IL-2693
619.2617	M	C	IL-2693
720.5499	F	C	IL-2693
678.417	F	C	IL-1513
548.9148	M	C	IL-1513
608.8224	M	C	IL-1513
566.6896	F	C	IL-711
709.4524	M	C	IL-1379
561.8932	F	C	IL-2438
568.7586	F	C	IL-2438
684.3419	M	C	IL-711
635.0615	M	C	IL-557
648.5101	F	C	IL-1513
700.7061	F	C	IL-3480
612.3962	F	C	IL-3480
533.3971	M	C	IL-3480
649.6387	F	C	IL-557
645.0305	F	C	IL-557
762.6827	F	C	IL-711
756.5697	F	C	IL-711
549.2355	F	C	AU8010
507.1491	F	C	AU8010
567.6484	F	C	AU8010
563.8343	F	C	AU8010

571.1995	M	C	AU8010
720.2678	M	C	IL-16296
550.8898	M	C	IL-1912,
616.9105	M	C	IL-57
635.7198	M	C	IL-57
574.2133	M	C	IL-1488
587.756	M	C	IL-1488
587.0977	M	C	IL-1488
770.9589	M	C	AU8016
783.1849	M	C	AU8016

Table S5. Genes under 95% confidence intervals under each QTL

Trait	QTL	Chr	CI %	Position (Mb)	Gene symbol	Merge strength
BV/TV and Tb.N	Trl7	11	95	112.1-113.6	Sox9	-
			90	113.6-116.6	Slc39a11	-
					Sstr2	-
					Cog1	-
					Fam104a	-
					D11Wsu47e	-
					Cpsf4l	-
					Cdc42ep4	-
					Sdk2	-
					Rpl38	-
					Ttyh2	-
					Dnaic2	-
					Kif19a	-
					Btbd17	-
					Gprc5c	-
					Cd300a	-
					ENSMUSG00000063193	-
					Cd300c	-
					Cd300ld	-
					AF251705	-
					Gm11710	-
					Gm11711	-
					Cd300lh	-
					Cd300e	-
					Rab37	-
					Cd300lf	-
					Slc9a3r1	-
					Nat9	-
					Tmem104	-
					Grin2c	-
					Fdxr	-
					Fads6	-
					Otop2	-
					Ush1g	-
					Otop3	-
					Hid1	-
					Cdr2l	-
					Ict1	-
					Atp5h	-
					Kctd2	-

		4933422H20Rik	-
		Slc16a5	-
		Armc7	-
		Nt5c	-
		Hn1	-
		Sumo2	-
		Nup85	-
		Gga3	-
		Mrps7	-
		Mif4gd	-
		Slc25a19	-
		Grb2	-
		2310067B10Rik	-
		Caskin2	-
		Tsen54	-
		Llgl2	-
		Myo15b	-
		Recql5	-
		Smim5	-
		Smim6	-
		Sap30bp	-
		Itgb4	-
		Galk1	-
		H3f3b	-
		Unk	14.29%
		Unc13d	-
		Wbp2	0.82%
		Trim47	-
		Trim65	-
		Mrpl38	-
		Fbf1	3.32%
		Acox1	10.65%
		Ten1	-
		Evpl	2.90%
		Srp68	-
		Galr2	-
		Exoc7	-
		Foxj1	6.35%
		Rnf157	1.36%
		Ubald2	6.00%
		Qrich2	5.13%
		Prpsap1	5.52%
		Sphk1	8.70%
		Ube2o	11.11%
50	116.6-116.7	**Aanat**	15.38%
		**Rhbdf2**	14.07%

					Cygb	11.11%
					Gm11744	1.99%
					1810032O08Rik	1.56%
					St6galnac2	-
			90	116.7-118.1	St6galnac1	-
					Mxra7	-
					Jmjd6	-
					Mettl23	2.53%
					Srsf2	4.35%
					Mfsd11	5.03%
					Mgat5b	-
					Sec14l1	-
					42622	-
					Gm11733	-
					Tnrc6c	-
					Tmc6	-
					Tmc8	-
					6030468B19Rik	-
					Gm20708	-
					Syngr2	-
					Tk1	-
					Afmid	-
					Birc5	-
					Tmem235	-
					Tha1	-
					Socs3	-
					Pgs1	-
					Dnah17	-
			95	118.1-118.3	Cyth1	-
					Usp36	-
Tb.Th	Trl8	4	95	110.8-113.05	Bend5	-
					Agbl4	-
					Spata6	-
					Slc5a9	-
			90	113.05-117.2	Skint8	-
					Skint7	-
					Skint1	-
					Skint4	-
					Skint3	-
					Skint9	-
					Skint2	-
					Skint10	-
					Skint6	-
					Skint5	-
					Skint11	-
					Trabd2b	-

Gm12830	-
Foxd2	-
Cmpk1	-
Stil	-
Tal1	-
Pdzk1ip1	-
Cyp4x1	-
Cyp4a12a	-
Cyp4a12b	-
Cyp4a14	-
Cyp4a10	-
Cyp4a31	-
Cyp4b1	-
Efcab14	-
Tex38	-
Atpaf1	-
Mob3c	-
Mknk1	-
Kncn	-
Dmbx1	-
Faah	-
Nsun4	-
Uqcrh	-
Lrrc41	-
Rad54l	-
Lurap1	-
Pomgnt1	-
Tspan1	-
Pik3r3	-
Mast2	-
Ipp	-
Tmem69	-
Gpbp1l1	-
Ccdc17	-
Nasp	-
Akr1a1	-
Prdx1	-
Mmachc	-
Ccdc163	-
Tesk2	-
Toe1	-
Mutyh	-
Hpdl	-
Zswim5	-
Urod	-
Hectd3	-

		Eif2b3	-
		Ptch2	-
		Btbd19	-
		Tctex1d4	-
		Plk3	-
		Rps8	-
		Kif2c	-
50	117.2-117.5	Gm1661	-
		Tmem53	-
		Rnf220	-
		Eri3	8.12%
90	117.5-125.54	Dmap1	18.27%
		Klf17	0.92%
		Slc6a9	6.73%
		Ccdc24	5.79%
		B4galt2	4.52%
		Atp6v0b	-
		Dph2	4.00%
		Ipo13	-
		Artn	2.08%
		St3gal3	14.18%
		Kdm4a	0.17%
		Ptprf	4.10%
		Hyi	-
		Szt2	-
		Med8	-
		Elovl1	-
		Cdc20	-
		Mpl	-
		Tie1	-
		2610528J11Rik	-
		Tmem125	-
		Cfap57	-
		Ebna1bp2	-
		Olfr1342	-
		Olfr1335	-
		Olfr1333	-
		Olfr1331	-
		Lao1	-
		Slc2a1	-
		Zfp691	-
		Ermap	-
		Ccdc23	-
		4930538K18Rik	-
		AU022252	-
		Lepre1	-

Cldn19	-
Ybx1	-
Ppih	-
Ccdc30	-
Ppcs	-
Zmynd12	-
Rimkla	-
AA415398	-
Foxj3	-
Guca2a	-
Guca2b	-
Hivep3	-
Edn2	-
Foxo6	-
Scmh1	-
Slfnl1	-
Ctps	-
Cited4	-
Kcnq4	-
Nfyc	-
Rims3	-
Exo5	-
Zfp69	-
Smap2	-
Col9a2	-
Zmpste24	-
Tmco2	-
Rlf	-
Gm12888	-
Gm12886	-
9530002B09Rik	-
Ppt1	-
Cap1	-
Mfsd2a	-
Mycl	-
Trit1	-
Bmp8b	-
Oxct2b	-
Ppie	-
Hpcal4	-
Nt5c1a	-
Heyl	-
Pabpc4	-
Bmp8a	-
Oxct2a	-
Macf1	-

Tb.Sp

					Ndufs5	-
					Akirin1	-
					Rhbdl2	-
					4933427I04Rik	-
					Mycbp	-
					Rragc	-
					3100002H09Rik	-
					Pou3f1	-
					Utp11l	-
					Fhl3	-
					Sf3a3	-
					Inpp5b	-
					Mtf1	-
					1110065P20Rik	-
					Yrdc	-
					Maneal	-
					Epha10	-
					Cdca8	-
					Rspo1	-
					Gnl2	-
					Dnali1	-
					Snip1	-
					Meaf6	-
					Zc3h12a	-
			95	125.54-126.52	Grik3	-
					Csf3r	-
					Mrps15	-
					Oscp1	-
					Lsm10	-
					Stk40	-
					Eva1b	-
					Sh3d21	-
					Thrap3	-
					Map7d1	-
					Trappc3	-
					Col8a2	-
					Adprhl2	-
					Tekt2	-
					Ago3	-
					Ago1	-
					Ago4	-
Tb.Sp	Trl9	5	95	99.8-101.6	Antxr2	-
					Prdm8	-
					Fgf5	-
					Bmp3	-
					Prkg2	-

		Rasgef1b	-
		A930011G23Rik	-
		Hnrnpd	-
		4930524J08Rik	-
		Hnrnpdl	-
		Enoph1	-
		Tmem150c	-
		Sec31a	-
		Lin54	-
		Cops4	-
		Plac8	-
		Coq2	-
		Hpse	-
		Helq	-
		Mrps18c	-
		Fam175a	-
		Agpat9	-
90	101.6-105.78	Nkx6-1	-
		Cds1	-
		Wdfy3	-
		Arhgap24	-
		Mapk10	-
		Ptpn13	-
		Slc10a6	-
		1700016H13Rik	-
		Aff1	-
		Klhl8	-
		Hsd17b13	-
		Hsd17b11	-
		Nudt9	-
		Gm17660	-
		Sparcl1	-
		Dspp	-
		Dmp1	-
		Ibsp	-
		Mepe	-
		Spp1	-
		Pkd2	-
		BC005561	-
		Zfp951	-
		Abcg3	-
50	105.78-106.14	Gbp8	5.88%
		Gbp9	15.12%
		Gbp4	5.00%
		Gbp6	9.01%
		ENSMUSG00000092021	12.35%

		Lrrc8b	26.15%
		Lrrc8c	9.08%
		Lrrc8d	4.02%
		Zfp326	1.00%
90	106.14-109.11	Barhl2	2.38%
		Zfp644	4.92%
		Hfm1	1.56%
		Cdc7	1.36%
		Tgfbr3	-
		Brdt	-
		Ephx4	-
		Lpcat2b	-
		Btbd8	-
		A830010M20Rik	-
		1700028K03Rik	-
		Glmn	-
		Rpap2	-
		Gfi1	-
		Evi5	-
		Ube2d2b	-
		Rpl5	-
		Fam69a	-
		Mtf2	-
		Tmed5	-
		Ccdc18	-
		Dr1	-
		Pigg	-
		Gm10419	-
		Pde6b	-
		Atp5k	-
		Mfsd7a	-
		Pcgf3	-
		Cplx1	-
		Gak	-
		Tmem175	-
		Dgkq	-
		Idua	-
		Slc26a1	-
		Fgfrl1	-
		Tmed11	-
		Vmn2r10	-
95	109.11-110.39	Crlf2	-
		5430403G16Rik	-
		4930522L14Rik	-
		Gm15446	-
		Zfp932	-

					Plcxd1	-
					Gtpbp6	-
					Zfp605	-
					Chfr	-
					Golga3	-
					Ankle2	-
					Pgam5	-
					Pxmp2	-
					Pole	-
					P2rx2	-
					Lrcol1	-
					Fbrsl1	-
Ct.Th	*Crl1*	4	95	3.4-4.0	Tmem68	-
					Tgs1	-
					Lyn	-
					Rps20	-
					Mos	-
					Plag1	-
					Chchd7	-
					Gm11808	-
			90	4.0-9.29	Sdr16c5	-
					Sdr16c6	-
					Penk	-
					Impad1	-
					Fam110b	-
					Ubxn2b	-
					Cyp7a1	-
					Sdcbp	-
					Nsmaf	-
					Tox	-
					Car8	-
					Rab2a	-
					Chd7	0.04%
			50	9.29-9.72	Clvs1	-
					Asph	0.07%
			90	9.72-11.7	Gdf6	-
					2610301B20Rik	-
					Plekhf2	-
					Ndufaf6	-
					Trp53inp1	-
					Ccne2	-
					Ints8	-
					Dpy19l4	-
					Esrp1	-
					1110037F02Rik	-
					Rad54b	-

					Fsbp	-
			95	11.7-11.8	Gem	-
					Cdh17	-
vBMD	Crl2	3	95	93.22-94.4	Flg2	-
					Flg	-
					Hrnr	-
					Rptn	-
					Tchh	-
					Tchhl1	-
					S100a11	-
					S100a10	-
					Tdpoz2	-
					Tdpoz1	-
					Gm5773	-
					Gm9117	-
					Gm9125	-
					Gm10696	-
					Gm5286	-
					Gm4778	-
					Them4	-
					Them5	-
					C2cd4d	-
					Rorc	-
			90	94.4-97.22	Lingo4	-
					Tdrkh	-
					Oaz3	-
					Mrpl9	-
					Riiad1	-
					Celf3	-
					Snx27	-
					Tuft1	-
					Selenbp2	-
					Cgn	-
					Pogz	-
					Psmb4	-
					Selenbp1	-
					Rfx5	-
					Pi4kb	-
					Zfp687	-
					Psmd4	-
					Pip5k1a	-
					Vps72	-
					Tmod4	-
					Scnm1	-
					Lysmd1	-
					Tnfaip8l2	-

Sema6c	-
Gabpb2	-
Mllt11	-
Cdc42se1	-
Gm128	-
Bnipl	-
Prune	-
Fam63a	-
Anxa9	-
Cers2	-
Setdb1	-
Arnt	-
Ctsk	-
Ctss	-
Hormad1	-
Golph3l	-
Ensa	-
Mcl1	-
Adamtsl4	-
Ecm1	-
Tars2	-
Rprd2	-
Prpf3	-
Mrps21	-
C920021L13Rik	-
Ciart	-
BC028528	-
Aph1a	-
Car14	-
Anp32e	-
Plekho1	-
Vps45	-
Otud7b	-
Mtmr11	-
Sf3b4	-
Sv2a	-
Bola1	-
Hist2h2ab	-
Hist2h2ac	-
Hist2h2be	-
Hist2h3c2	-
Hist2h2aa1	-
Hist2h2aa2	-
Hist2h3c1	-
Hist2h4	-
Hist2h3b	-

		Hist2h2bb	-
		Fcgr1	-
		BC107364	-
		Hfe2	-
		Txnip	-
		Gm16253	-
		Polr3gl	-
		Ankrd34a	-
		Lix1l	-
		Rbm8a	-
		Pex11b	-
		Itga10	-
		Ankrd35	-
		Pias3	-
		Nudt17	-
		Polr3c	0.39%
		Rnf115	1.19%
		Cd160	-
		Pdzk1	-
		Gpr89	-
		Gja8	-
		Gja5	-
50	97.2-97.4	Acp6	1.82%
		Bcl9	3.54%
		Chd1l	-
90	97.4-103.1	Fmo5	17.61%
		Prkab2	15.58%
		Pde4dip	7.24%
		Sec22b	20.78%
		Notch2	-
		Adam30	-
		Reg4	-
		Hmgcs2	-
		Phgdh	-
		Zfp697	-
		Gm4450	-
		Gm10681	-
		Hsd3b5	-
		Hsd3b2	-
		Hsd3b3	-
		Hsd3b6	-
		Hsd3b1	-
		Hao2	-
		Wars2	-
		Tbx15	-
		Wdr3	-

		Spag17	-
		Gdap2	-
		Fam46c	-
		Man1a2	-
		Vtcn1	-
		Trim45	-
		Ttf2	-
		Cd101	-
		Ptgfrn	-
		Igsf3	-
		Atp1a1	-
		Mab21l3	-
		Slc22a15	-
		Nhlh2	-
		Casq2	-
		Vangl1	-
		Ngf	-
		Tspan2	-
		Tshb	-
		Sycp1	-
		Nr1h5	-
		Sike1	-
		Csde1	-
		Nras	-
		Ampd1	-
95	103.1-104.3	Dennd2c	-
		Bcas2	-
		Trim33	-
		Syt6	-
		Olfml3	-
		Hipk1	-
		Dclre1b	-
		Ap4b1	-
		Bcl2l15	-
		Ptpn22	-
		Rsbn1	-
		Phtf1	-
		Magi3	-

Genes under the QTLs for the cortical and trabecular traits. Light blue is the 95% CI, blue is the 90% CI, and red is the 50% CI. "Merge Strength" refers to the proportion of merge logP values constricted to the region of the specific gene. Note that for T*rl*7 which is common to BV/TV and Tb.N, the average values between the two are provided.
